# A geometrical multi-scale numerical method for coupled hygro-thermo-mechanical problems in photovoltaic laminates

**DOI:** 10.1007/s00466-016-1271-5

**Published:** 2016-02-18

**Authors:** P. Lenarda, M. Paggi

**Affiliations:** Research Unit on Multi-scale Analysis of Materials (MUSAM), IMT Institute for Advanced Studies Lucca, Piazza San Francesco 19, 55100 Lucca, Italy

**Keywords:** Thermo-visco-elasticity, Moisture diffusion, Cohesive zone model, Geometrical multiscale model, Photovoltaics

## Abstract

A comprehensive computational framework based on the finite element method for the simulation of coupled hygro-thermo-mechanical problems in photovoltaic laminates is herein proposed. While the thermo-mechanical problem takes place in the three-dimensional space of the laminate, moisture diffusion occurs in a two-dimensional domain represented by the polymeric layers and by the vertical channel cracks in the solar cells. Therefore, a geometrical multi-scale solution strategy is pursued by solving the partial differential equations governing heat transfer and thermo-elasticity in the three-dimensional space, and the partial differential equation for moisture diffusion in the two dimensional domains. By exploiting a staggered scheme, the thermo-mechanical problem is solved first via a fully implicit solution scheme in space and time, with a specific treatment of the polymeric layers as zero-thickness interfaces whose constitutive response is governed by a novel thermo-visco-elastic cohesive zone model based on fractional calculus. Temperature and relative displacements along the domains where moisture diffusion takes place are then projected to the finite element model of diffusion, coupled with the thermo-mechanical problem by the temperature and crack opening dependent diffusion coefficient. The application of the proposed method to photovoltaic modules pinpoints two important physical aspects: (i) moisture diffusion in *humidity freeze* tests with a temperature dependent diffusivity is a much slower process than in the case of a constant diffusion coefficient; (ii) channel cracks through Silicon solar cells significantly enhance moisture diffusion and electric degradation, as confirmed by experimental tests.

## Introduction and motivations

Photovoltaic modules are composite structures obtained by laminating layers of various materials. Some have the role to guarantee protection from the environment by a suitable sealing of the device, others have specific electric features to produce energy. The durability of these devices is a serious concern due to fracture events promoted by the mismatch between the thermo-mechanical properties of the material constituents, often amplified by the severe working conditions they are exposed to. Moreover, their modelling is changelling and it requires a multi-physics framework to gain an accurate picture of their overall working conditions, performance and degradation [1].

**Fig. 1:**
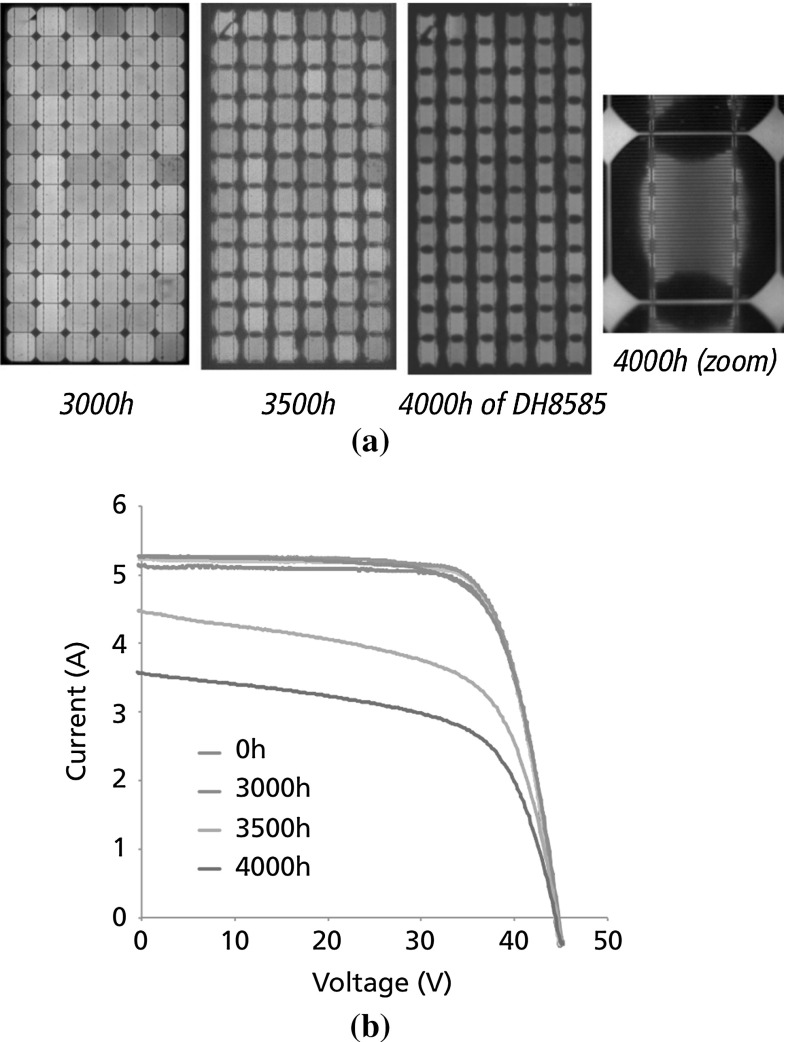
Electric degradation during the *damp heat* test ($T=85^{\circ }$, $RH=85\,\%$). Electroluminescence images show electric degradation under the form of dimmer electrically inactive areas (adapted from [2]) **a** moisture effects, **b** I–V *curves*

Typical photovoltaic (PV) modules are laminates made of a thick glass superstrate, an encapsulating polymer (usually ethylene vinyl acetate, EVA), a layer of interconnected Silicon solar cells separated by few centimeters of EVA in their plane, another layer of EVA, and finally a polymeric protective backsheet. EVA provides protection of cells and interconnections but it is permeable to moisture, which diffuses from the backsheet and percolates along the surface of the solar cells. In turn, moisture induces chemical oxidation of the grid line deposited on the surface of the solar cells, giving rise to electrically inactive areas and power-loss. This phenomenon has been reported in *damp heat* tests in [2] prescribed by the international qualification standards [4], where PV modules were exposed to a very aggressive environment at constant $85\,^{\circ }$C temperature and $85\%$ of air humidity. In particular, it has been shown a progressive increase of dimmer areas in time in the electroluminescence (EL) images starting from the edges of the solar cells towards their center (see Fig. 1a). Correspondingly, the current-voltage of the PV module degrades, with a significant power-loss (see Fig. 1b).

**Fig. 2:**
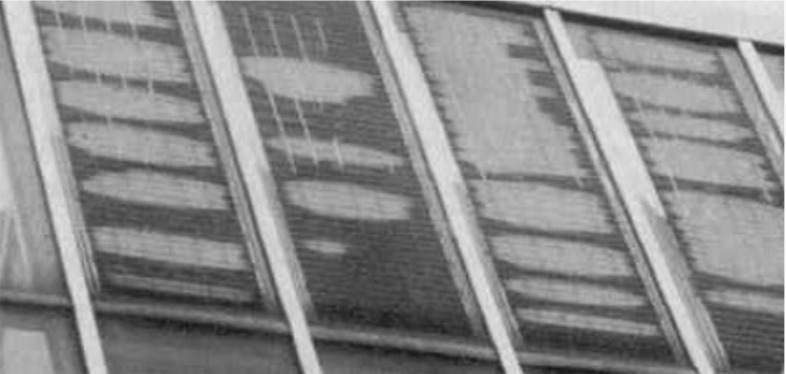
Example of delamination of the glass from the solar cells, adapted from [3]

For these reasons, an accurate modelling of the EVA is crucial to determine the lifetime of a PV module, moisture diffusion, chemical reactions induced by moisture, as well as its reduction of cohesive energy promoting delamination of the layers (see Fig. 2).

**Fig. 3:**
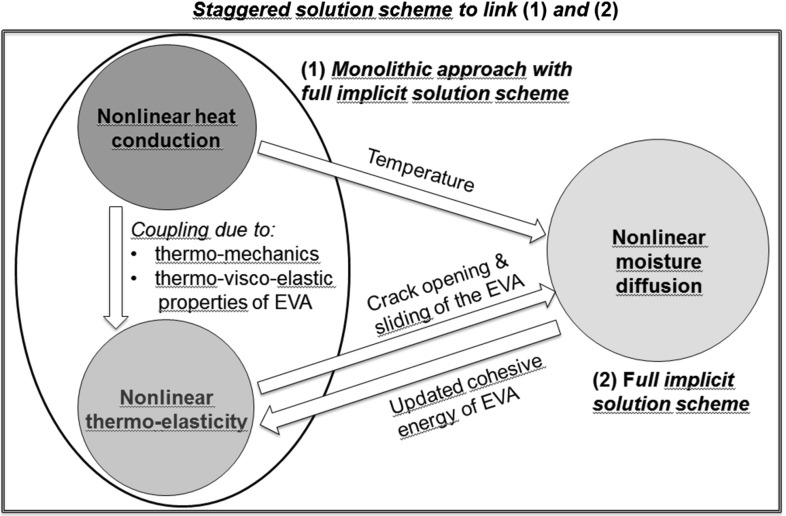
Multi-physics modelling showing the proposed solution schemes and the interactions between the various fields

The EVA polymer displays a strong thermo-visco-elastic constitutive response, as experimentally reported in [5, 6], with a variation of the elastic modulus of up to three orders of magnitude depending on temperature. Generalized Maxwell rheological models used so far generally provide exponential type relations for the relaxation modulus and, in order to approximate the experimentally observed power-law trend, a huge number of elements (and thus of model parameters) has to be taken into account. To significantly simplify the task of parameters identification, modelling the visco-elastic behaviour via fractional derivatives has been proved to be very effective [8–10]. For rheologically complex polymers as EVA, whose microstructure changes with temperature, the fractional calculus formulation in [11] allows the use of only two temperature dependent parameters for its complete description.

Another complexity regards the moisture diffusion properties of EVA, strongly temperature dependent as experimentally reported in [12], which implies coupling between moisture and temperature fields. In return, moisture is degrading the cohesive energy of the EVA encapsulant, promoting decohesion of the backsheet or separation of the glass cover from the solar cells [13, 14]. This mathematically corresponds to coupling between the mechanical and the diffusive fields, with a further acceleration of moisture diffusion and degradation as reported in [15], which is a feedback coupling from diffusion to mechanics.

A proper modelling of these coupled nonlinear phenomena, whose conceptual interactions are sketched in Fig. 3, requires a comprehensive computational framework where coupled thermo-elastic and heat conduction problems are accurately solved at the module level in the three-dimensional space. Then, moisture diffusion inside the EVA layers has to be simulated by considering the dependency on the temperature and the thermo-elastic fields via the diffusive constitutive equations. So far, the state-of-the-art simulations on moisture diffusion in [12] consider the EVA layer only and treat diffusion as a one dimensional problem without updating the diffusivity of the material based on the actual temperature of the system. The former approximation of considering moisture diffusion as a pure 1D problem fails when channel cracks in Silicon solar cells are present, since they can also be a source of moisture percolation from the backsheet to the front side of the solar cells. The latter assumption of using a constant diffusivity (uncoupling with the thermal field) is an approximation valid only in the steady state temperature regime. While this is the case of the *damp heat* test, its validity in the case of a cyclic variation of temperature from $-40$ up to $85\,^{\circ }$C as in the *humidity freeze* test is highly questionable.

**Fig. 4:**
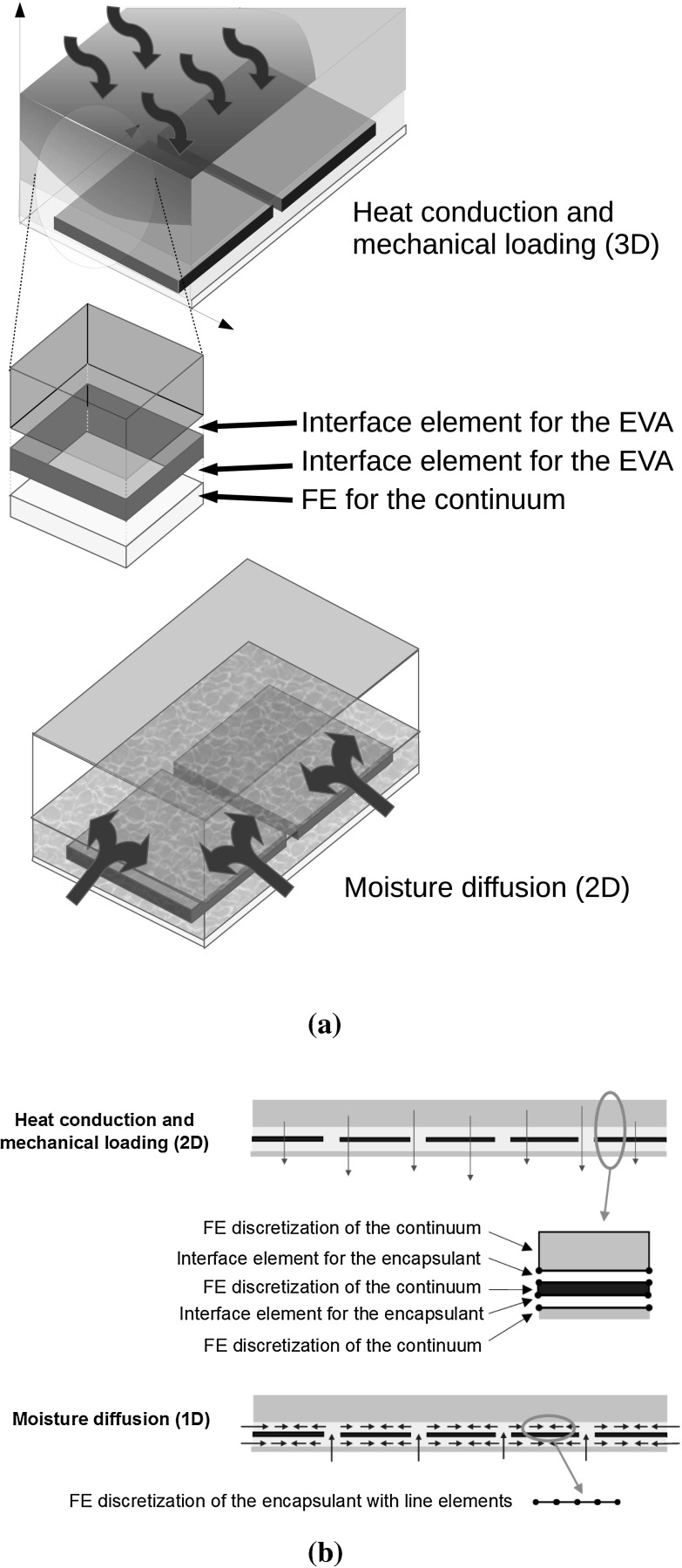
Proposed finite element models **a** 3D laminate models, **b** 2D cross-section models

To shed light into the above issues, and provide a comprehensive physical modelling and computational framework for the study of these phenomena, a geometrical multiscale approach is herein proposed by following the seminal work in [16] for biophysical systems. Starting from the evidence that moisture diffusion takes place in a physical domain with a lower dimension with respect to that of the thermo-mechanical and heat conduction problems, two different finite element models are used in parallel. The coupled thermo-mechanical and heat conduction problems are solved in the three-dimensional setting (or in the two-dimensional one in the case of a cross-section of the PV module). As a further simplification, the EVA encapsulant layers are modelled as zero-thickness interfaces, whose thermo-visco-elastic constitutive response is taken into account by a novel thermo-viscoelastic cohesive zone model. As compared to other cohesive zone model formulations available in the literature [17, 18], the present formulation is based on fractional calculus and it is able to simulate rheologically complex materials.

The thermo-mechanical problem, which is much faster than moisture diffusion, is solved first via a fully implicit solution scheme in space and time, see Fig. 2. Temperature and relative displacements computed in the Gauss points along the encapsulant interfaces are then projected to the nodes of another finite element model specific for the solution of moisture diffusion. This second model is used to discretize the domain where moisture diffusion takes place. In particular, it is represented by the mid-surfaces of the encapsulant layers and of the channel cracks through Silicon, see Fig. 4.

This article is structured as follows. In Sect. 2, the variational framework for thermo-mechanics and heat conduction for the layers is presented, along with the interface model for the thermo-visco-elastic encapsulant, as well as for moisture diffusion. The weak forms of the partial differential equations are established in Sect. 3 and the finite element discretizations are presented in Sect. 4. Details on the proposed numerical solution scheme are provided in Sect. 5 and numerical applications to photovoltaics and comparison with experiments are collected in Sect. 6. Conclusions and an overview of future perspectives of research complete the study.

## Variational framework

In this section, the variational framework describing moisture diffusion and the thermo-visco-elastic response of a laminate made of linear elastic homogeneous and isotropic layers separated by polymeric thermo-visco-elastic laminae is presented. In these laminates, moisture diffusion takes place in the polymeric layers, which progressively percolates from the free edges and from the permeable backsheet towards the centre of the solar cells. EVA layers used to protect Silicon solar cells are permeable to moisture, which is one of the major concerns for the degradation of the electrical output of the PV module over time. Due to moisture diffusion, the adhesive properties of EVA progressively degrade and the corresponding layers may experience a lack of cohesion leading to separation of the backsheet from the Silicon cells, or between the Silicon cells from the glass superstrate. In order to efficiently simulate cohesive degradation of EVA and delamination, we propose to treat the polymeric layers as zero-thickness internal interfaces with suitable traction-separation relations accounting for their thermo-visco-elastic properties.

Let the laminate occupy a volume $$\varOmega = \cup ^n_{m=1} R(m) \subset \mathbb {R}^3 $$ in the reference undeformed configuration, where each layer is geometrically identified by $$R(m)=[0, a] \times [0, b] \times [z_{m-1}, z_m]$$, with $$z_0=0$$, $$z_m=z_{m-1}+h_m$$, where $$a,b\gg h_m$$ for $$m=1,\dots , n$$. Moreover, let model a generic polymeric layer of thickness *h* between laminae $$R(p)=R_{-}$$ and $$R(p+1)=R_{+}$$$$(1\le p\le n)$$ as a plane surface $$S(p)=(x_1,x_2,z_p) \ : 0\le x_1 \le a, 0\le x_2\le b\}$$, see Fig. 4.

The position of each material point inside $$\varOmega $$ is identified by the coordinate vector $$\mathbf {x}=(x_1,x_2,x_3)^{\mathbf {T}}$$ in a three-dimensional cartesian orthonormal frame $$\{\mathbf {e}_1, \mathbf {e}_2 , \mathbf {e}_3 \}$$. Let $$u_I(x_1,x_2,x_3,t)$$$$(I=1, 2, 3)$$ and $$\theta (x_1,x_2,x_3,t)=T(x_1,x_2,x_3,t)-T_0$$ be the displacement field and temperature variation from a reference one, $$T_0$$, inside the material during the time interval $$0\le t\le t_f$$. The index *I* is used to denote the component of the displacement field along the corresponding coordinate.

### Thermo-mechanical formulation of the layers

The global dynamics of each material layer *R*(*m*) obeys the equations of coupled linear isotropic thermo-elasticity (see e.g. [19, 20]). The Cauchy thermal stress tensor is defined for each layer *m* as:

1 $$\begin{aligned} \sigma _{IJ}=C^{m}_{IJKL} \varepsilon _{KL} - \beta ^{m} \theta \delta _{IJ},\quad 1\le I,J,K,L \le 3 \end{aligned}$$

where the Einstein summation notation has been adopted. Here, $C^{m}_{IJKL}$ is the fourth order elastic constitutive tensor, and $\beta ^{m}$ is the coupling thermal factor related to the thermal expansion coefficient $\alpha ^{m}$. The infinitesimal strain tensor is:

2 $$\begin{aligned} \varepsilon _{IJ}=\dfrac{1}{2} \left( \dfrac{ \partial u_I }{ \partial x_J }+ \dfrac{ \partial u_J }{ \partial x_I } \right) ,\quad 1\le I,\quad J\le 3 \end{aligned}$$

The balance of linear momentum is given by:

3 $$\begin{aligned} \begin{aligned}&\rho ^{m} \dfrac{\partial ^2 u_I}{\partial t^2} - \dfrac{\partial }{\partial x_J } \left( C^{m}_{IJKL} \varepsilon _{KL} -\beta ^{m} \theta \delta _{IJ} \right) =0 \\&\quad \text {in} \ R(m) \times [0,t_f] , \quad I=1,2,3 \end{aligned} \end{aligned}$$

where $\rho ^m$ is the density of the *m*-th material.

Let $q_I$ be the heat flux inside each layer *R*(*m*), and assume that it is related to the temperature variation $\theta$ by the Fourier law:

4 $$\begin{aligned} q_I=-k^{m} \dfrac{\partial \theta }{ \partial x_I},\quad 1\le I\le 3 \end{aligned}$$

where $k^{m}>0$ is the thermal conductivity of the *m*-th material. Hence, the heat transfer partial differential equation is given by:

5 $$\begin{aligned} k^{m} \nabla ^2 \theta = \rho ^{m} c^{m} \dfrac{ \partial \theta }{\partial t} + T_0 \beta ^{m} \dfrac{\partial {\varepsilon _{KK}}}{\partial t} \quad \text {in} \quad R(m) \times [0,t_f] \end{aligned}$$

where $c^{m}$ is the heat capacity of the *m*-th material.

### Thermo-visco-elastic polymeric interfaces

Under the assumption of replacing each polymeric layer by a zero-thickness imperfect interface, we allow $u_I$ and the temperature $\theta$ to be discontinuous across the interface separating $R_-$ from $R_+$. We therefore define the jumps on *S*(*p*) as:

6a $$\begin{aligned}{}[[ u_I ]](x_1,x_2)&=u^+_I(x_1,x_2)-u^-_I(x_1,x_2),\quad 1\le I\le 3, \end{aligned}$$

6b $$\begin{aligned}{}[[ \theta ]](x_1,x_2)&=\theta ^+(x_1,x_2)-\theta ^-(x_1,x_2) \end{aligned}$$

where $u^{\pm }_I(x_1,x_2)=u_I(x_1, x_2, z^{\pm }_p)$ and $\theta ^{\pm }_I(x_1,x_2)=\theta _I(x_1, x_2, z^{\pm }_p)$. The average temperature across the interface is given by:

7 $$\begin{aligned} \left\langle \theta \right\rangle (x_1,x_2)= \dfrac{1}{2} \left[ \theta ^+(x_1,x_2)+\theta ^-(x_1,x_2) \right] \end{aligned}$$

Quantities $[[u_I]]$ and $[[\theta ]]$ play the role of internal variables describing the debonding process along the interface containing *S*(*p*) during time $[0, t_f]$.

Hence, the coupled thermo-elastic model given in the previous section for the continuum layers is enriched by adding the presence of a cohesive traction field and a heat flux normal to *S*(*p*). The relations between those fields and $[[ u_I ]]$, $[[ \theta ]]$ are provided by the constitutive relations for the interface. By assuming the continuity of the traction vector components $t_I$, the in-plane sliding and out-of-plane tearing components of the traction vector read:

8 $$\begin{aligned} t_I={\left\{ \begin{array}{ll} K_I \left( t, \left\langle \theta \right\rangle \right) [[u_I]], &{} \quad \text {if} \ [[u_I]] \in J_I\\ 0, &{} \quad \text {if}\ [[u_I]] \notin J_I \end{array}\right. } \end{aligned}$$

where $I=1,2$ and $J_I=(-\delta ^c_I, +\delta ^c_I)$, while the opening traction component is:

9 $$\begin{aligned} t_3={\left\{ \begin{array}{ll} \epsilon [[u_3]] , &{} \quad \text {if} \ [[u_3]] < 0 \\ K_3 \left( t, \left\langle \theta \right\rangle \right) [[u_3]], &{} \quad \text {if} \ [[u_3]] \in J_3\\ 0, &{} \quad \text {if}\ [[u_3]] \notin J_3 \quad \text {and} \quad [[u_3]] \ge 0 \end{array}\right. } \end{aligned}$$

where $$J_3=(0, \delta ^c_3)$$.

**Fig. 5:**
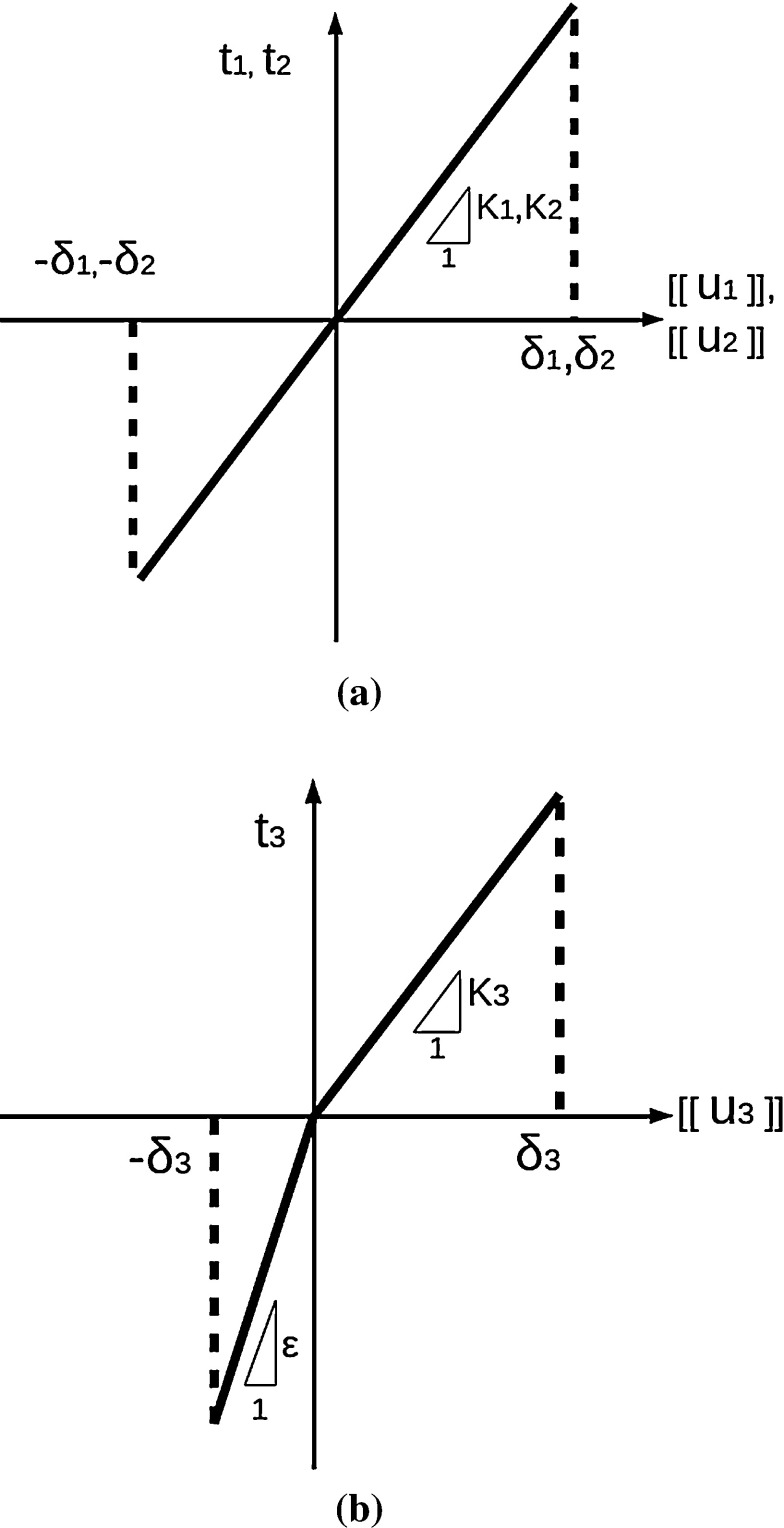
Cohesive traction-separation relations. **a** Sliding and tearing fracture modes. **b** Opening fracture mode

Equation (9) corresponds to a tension cut-off traction-separation curve, suggesting that the interface is not able to transfer tractions when the critical opening displacement $\delta ^c_3$ is overcome. A similar brittle behavior is assumed for sliding and tearing fracture modes (see Fig. 5). In compression, a penalty formulation with penalty parameter $\epsilon$ is adopted as in [7].

In order to obtain a structural response of the interface equivalent to that of the real EVA layers, the modulus $K_3$ of the zero-thickness interface is related to the actual stiffness of the layer in the direction $\mathbf {n}$, i.e., it can be evaluated as the ratio between the EVA Young’s modulus, $E_{\text {EVA}}$, and its thickness, $h_{\text {EVA}}$, i.e., $K_3=E_{\text {EVA}}/h_{\text {EVA}}$. Similarly, the shear response is matched by selecting $K_1=K_2=E_{\text {EVA}}/\left[ 2(1+\nu _{\text {EVA}})\right]$.

Since polymers have a thermo-visco-elastic constitutive behavior, the stiffnesses $K_I$ have to depend both on the average temperature $\left\langle \theta \right\rangle$ and time *t*. Instead of using a Prony series representation, a fractional calculus approach [8, 9] is herein adopted to synthetically characterize those dependencies. This approach has been proved in [11] to be very effective for parameters identification. Accordingly, $E_{\mathrm {EVA}}$ is defined as follows:

10 $$\begin{aligned} E_{\mathrm {EVA}}(t,T)=\dfrac{a(T) \ h(t,T)^{-\alpha (T)}}{\varGamma (1-\alpha (T))} \end{aligned}$$

for functions $0<a, \alpha <1$ and $\varGamma (t)$ is the Euler gamma function

11 $$\begin{aligned} \varGamma (t)=\int ^{\infty }_0 e^{-x} x^{t-1}\mathrm {d}x \end{aligned}$$

Function *h*(*t*, *T*) is a history dependent function of time and temperature used to model thermo-rheologically complex materials where the principle of time-temperature superposition does not apply. This is due to a modification of the internal microstructure of the polymer driven by a temperature change above a threshold. Hence, *h*(*t*, *T*) is equal to the current time *t* minus the time $t_0$ corresponding to such a microstructure modification.

At the interface, we remark that cohesive tractions are continuous and opposing to each other, viz.

12 $$\begin{aligned} \begin{aligned} t_I&= t^+_I=C^{(+)}_{IJKL}\varepsilon ^+_{KL}|_{x_3=z^+_p}n_J= -C^{(-)}_{IJKL}\varepsilon ^-_{KL}|_{x_3=z^-_p}n_J\\&=-t^-_I \end{aligned} \end{aligned}$$

As far as heat conduction is concerned, we assume that the heat flux across the interfaces is oriented along the direction orthogonal to the surface *S*(*p*), which is a reasonable approximation for thin polymeric layers. Hence, $q_1=q_2=0$ and $q=q_3$ is given by

13 $$\begin{aligned} q={\left\{ \begin{array}{ll} - \kappa _0 \left( 1- \dfrac{[[u_3]]}{\delta ^c_3} \right) \left\langle \theta \right\rangle &{} \quad \text {if}\;[[u_3]] \in J_3\\ 0 &{} \quad \text {if}\; [[u_3]] \notin J_3 \end{array}\right. } \end{aligned}$$

where $\kappa _0$ is the thermal conductivity of the interface without decohesion, i.e., for $[[ u_3 ]]=0$. Note that the heat flux is assumed to be a decreasing function of the normal gap, in order to account for partial heat transfer in the case of a damaged interface, see also [21].

Continuity of heat flux at the interface is preserved, viz.

14 $$\begin{aligned} \begin{aligned} q&= q^+_3(x_1,x_2)=-k^{(+)} \dfrac{\partial \theta ^+}{\partial x_3}|_{x_3=z^+_p}= k^{(-)} \dfrac{\partial \theta ^-}{\partial x_3}|_{x_3=z^-_p} \\&=-q^-_3(x_1,x_2). \end{aligned} \end{aligned}$$

### Moisture diffusion along polymeric interfaces

Durability tests of PV modules inside a climate chamber are characterized by temperature and moisture dependent on time according to specified ramps. In these composites, moisture diffusion takes place along the layers of the polymeric encapsulant, or along channel cracks in Silicon. Since their thickness is very small, it is possible to neglect moisture flux in the direction orthogonal to the EVA layers. Under these assumptions, moisture diffusion can be modelled as a diffusion process taking place over the mid-surface of a generic encapsulant layer, which corresponds to *S*(*p*).

Hence, the aim of the numerical method reduces to simulate and predict diffusion of water content $c(x_1,x_2,t)$ along the encapsulant mid-surface *S*(*p*) for each point and time.

The following initial and boundary value problem can be considered, where an imposed water content $c^*$ is imposed on the boundary:

15 $$\begin{aligned} {\left\{ \begin{array}{ll} \dfrac{\partial c}{\partial t}(x_1,x_2,t)-D \nabla ^2 c(x_1,x_2,t)=0 &{} \quad \! \text {in} \; S(p) \times [0,t_f] \\ c(x_1,x_2,0)=0&{} \quad \! \text {in} \; S(p)\\ c(x_1,x_2,t)=c^*&{} \quad \! \text {in} \; \partial S(p) \times (0, t_f] \end{array}\right. } \end{aligned}$$

where *D* is the encapsulant diffusivity.

In addition to a reduced dimensionality of the domain where moisture diffusion takes place with respect to thermo-elasticity, it is also remarkable to notice that these physical problems are characterized by very different time scales. The characteristic velocity of thermal diffusion is in fact ruled by the ratio $k^{m}/(\rho ^{m} c^{m})$, while that of moisture diffusion is related to the diffusivity *D*. Considering characteristic values for EVA, the ratio between the velocities of the two phenomena is:

$$\begin{aligned} \left( k^{m}/ (\rho ^{m} c^{m} ) \right) / D \approx 10^6, \qquad \forall m=1, \dots ,n \end{aligned}$$

so that heat transfer is about six order of magnitude faster than moisture diffusion. From this observation, we can state that moisture diffusion is dependent on heat transfer and not viceversa. Hence, the diffusivity of the encapsulant has to be considered as temperature dependent and, based on the experimental evidence [12], of Arrhenyus type:

16 $$\begin{aligned} D={\left\{ \begin{array}{ll} A \, \text {exp} \left( -\dfrac{E_a}{\left\langle \theta \right\rangle R} \right) , &{} \quad \text {if} \ [[ u_3 ]] \le \delta ^c_3 \\ A \, \text {exp} \left( -\dfrac{E_a}{\left\langle \theta \right\rangle R} \right) \dfrac{ [[ u_3 ]] }{\delta ^c_3}, &{}\quad \text {if} \ [[ u_3 ]] > \delta ^c_3 \end{array}\right. } \end{aligned}$$

In order to take into account the effect of a possible debonding of the encapsulant, which would enhance moisture diffusion, *D* is assumed to be a linear increasing function of the normal gap $[[u_3 ]]$, for $[[ u_3 ]]$ larger than $\delta _3^c$.

Due to the very different time scales of the diffusion processes, a staggered solution scheme is proposed, where the average temperature and the crack opening computed from the solution of the coupled thermo-mechanical problem are passed as input to the diffusion process by a suitable update of the value of *D*.

## Weak forms

The partial differential equations governing the dynamic equilibrium of the body, Eq. (3), and heat conduction, Eq. (5), for each layer *R*(*m*) $(m=1, \dots , n)$ and the constitutive relations for the interfaces, Eqs. (8), (9) and (13) for each *S*(*p*), define an initial boundary value problem describing the debonding of a thermo-mechanical layered PV panel with thermo-visco-elastic polymeric interfaces.

Let $t^*_I$ and $u^*_I$ be the surface traction and the prescribed boundary displacement such that:

$$\begin{aligned} \begin{array}{lll} &{}C_{IJKL} \varepsilon _{KL}(u_M)n_J =t^*_I &{}\quad \text {in} \; \partial _N R^u \times (0, t_f] , \\ &{}u_I =u^*_I &{}\quad \text {in} \; \partial _D R^u \times (0, t_f] \end{array} \end{aligned}$$

and $q^*=q^*_I n_I$, and $\theta ^*$ be the imposed normal heat flux and the imposed temperature at the boundaries such that:

$$\begin{aligned} \begin{array}{lll} &{}\dfrac{ \partial \theta }{\partial n }=q^* &{}\quad \text {in} \ \partial _N R^{\theta } \times (0, t_f] , \\ &{}\theta _I=\theta ^*_I &{}\quad \text {in} \ \partial _D R^{\theta } \times (0, t_f] \end{array} \end{aligned}$$

where the indices $\partial _D$ and $\partial _N$ denote the Dirichlet and the Neumann portions of the boundary $\partial R$.

The weak form corresponding to Eq. (3) is obtained by multiplying it by a virtual displacement $\delta v_I$ having a virtual gap $[[ \delta v_I ]]$ along *S*(*p*) and by integrating the result on each domain *R*(*m*). After applying the divergence theorem as customary and by dropping the index *m* to simplify notation, we obtain:

17 $$\begin{aligned} \begin{aligned}&\int _R \rho \dfrac{ \partial ^2 u_I }{ \partial t^2 } \delta v_I \mathrm {d}V + \int _R C_{IJKL} \varepsilon _{KL}\delta \varepsilon _{IJ} \mathrm {d}V \\&\quad -\int _R \beta \delta \varepsilon _{IJ} \delta _{IJ} \theta \mathrm {d}V= \int _{\partial _N R^u} t^*_I \delta v_I \mathrm {d}A\\&\quad + \int _{S(p)} t_I[[ \delta v_I ]] \mathrm {d}A \end{aligned} \end{aligned}$$

where $\delta _{IJ}$ is the Kronecker operator, and $\delta$ denotes a virtual variation of the variable to which is applied.

Analogously, the weak form corresponding to Eq. (5) is obtained by multiplying it by a test function $\delta \theta$ having a gap $[[ \delta \theta ]]$ on *S*(*p*) and by integrating the result on each domain *R*(*m*). After some calculation and dropping the index *m* to simplify notation, we get:

18 $$\begin{aligned} \begin{aligned}&\int _R k \dfrac{\partial \theta }{\partial x_I } \dfrac{\partial \theta }{\partial x_I} \mathrm {d}V + \int _R \rho c \dfrac{\partial \theta }{\partial t} \delta \theta \mathrm {d}V \\&\quad + \int _R \beta \dfrac{\partial \varepsilon _{IJ}}{\partial t} \delta _{IJ} \delta \theta \mathrm {d}V+\int _{\partial _N R^{\theta }} q^* \delta \theta \mathrm {d}A\\&\quad + \int _{S(p)} q[[ \delta \theta ]]\mathrm {d}A=0 \end{aligned} \end{aligned}$$

As far as moisture diffusion is concerned, the corresponding weak form is constructed by multiplying Eq. (15) by a test function $\delta c$. After integration by parts we have that the concentration $c(x_1, x_2,t)$ solves the following equation for all the admissible $\delta c$ and $\forall t \in [0, t_f]$:

19 $$\begin{aligned} \int _{S(p)} D \dfrac{\partial c }{ \partial x_I } \dfrac{\partial \delta c }{ \partial x_I } \mathrm {d}A + \int _{S(p)} \delta c \dfrac{\partial c}{\partial t} \mathrm {d}A=0. \end{aligned}$$

## Finite element discretization

### Discretized weak forms for the thermo-elastic and heat conduction problems

According to the finite element method, the domain *R* is discretized into a finite number of bulk $R_e$ and interface $\tilde{S}_e$ elements so that:

20 $$\begin{aligned} R \approx \bigcup _e R_e \cup \bigcup _e \tilde{S}_e \end{aligned}$$

We also introduce for the purpose of numerical integration the mid-plane surface $S(p) \approx \cup _e S_e$, where $S_e$ is the middle surface of each interface element $\tilde{S}_e$.

The class of interface elements considered here consists of two surface elements coincident with the facets of the bulk elements used to discretize the continuum that are bricks or tetrahedra. For consistency between interfaces and bulk, the same order of interpolation is used. In the case of 2D simulations on cross-sections of a laminate, the present formulation still holds, provided that bulk elements are represented by quadrilateral or triangular plane strain finite elements and interface elements are given by two opposing lines. Again, the same interpolation order has to be used.

By introducing the shape functions, the finite element approximation for the bulk reads:

$$\begin{aligned}&U_{K}(x_1,x_2,x_3,t)=\sum ^{N(e)}_{a=1} \varPhi _a(\xi _1, \xi _2 , \xi _3)U_{aK}(t),\quad 1\le K\le 3 \\&\varTheta (x_1,x_2,x_3,t)=\sum ^{N(e)}_{a=1} \varPhi _a(\xi _1, \xi _2 , \xi _3)\varTheta _a(t) \end{aligned}$$

being $\{ \varPhi _a(\xi _1, \xi _2 , \xi _3) \}^{N(e)}_{a=1}$ defined in the natural reference system $-1 \le \xi _1 , \xi _2 , \xi _3 \le +1$, where *N*(*e*) is the number of element nodes, which is equal to 8 for a 3*D* linear brick element, or 4 for a 2*D* linear 4-node plane strain element.

Similarly, for the interface elements, the gaps are approximated as:

$$\begin{aligned}{}[[U_{J}]](x_1,x_2,t)&=\sum ^{S(e)}_{a=1} \sum ^{2 S(e)}_{b=1} \varPsi _a(\xi _1, \xi _2) \Delta _{aJbK} U_{bK}(t) ,\\&\;\,\quad 1\le J\le 3\\ [[ \varTheta ]](x_1,x_2,t)&=\sum ^{S(e)}_{a=1} \sum ^{2 S(e)}_{b=1} \varPsi _a(\xi _1, \xi _2) \Delta _{ab} \varTheta _b(t) \end{aligned}$$

where the shape functions $\{ \varPsi _1(\xi _1, \xi _2 ) \}^{S(e)}_{a=1}$ are defined along the mid-surface plane in the natural reference system $-1 \le \xi _1 , \xi _2 \le +1$ and 2*S*(*e*) is the number of nodes of the interface element which is equal to 8 for a 3*D* interface element compatible with bricks, or 4 for a 2*D* interface element compatible with plane strain elements. The nodal displacement vector is:

$$\begin{aligned}&(U_{11},U_{12},U_{13},\dots ,U_{2S(e)1},U_{2S(e)2},U_{2S(e)3})^{\mathrm {T}}\\&\quad =\left( U^{+}_{11},U^{+}_{12},U^{+}_{13},\dots ,U^{+}_{S(e)1}, U^{+}_{S(e)2},U^{+}_{S(e)3},\right. \\&\qquad \quad \left. U^{-}_{11},U^{-}_{12},U^{-}_{13},\dots ,U^{-}_{S(e)1}, U^{-}_{S(e)2},U^{-}_{S(e)3}\right) ^{\mathrm {T}} \end{aligned}$$

and the temperature vector is

$$\begin{aligned}&(\varTheta _1,\dots ,\varTheta _{2S(e)})^{\mathrm {T}}\\&\quad = \left( \varTheta ^{+}_1,\dots ,\varTheta ^{+}_{S(e)}, \dots ,\varTheta ^{-}_1,\dots ,\varTheta ^{-}_{S(e)}\right) ^{\mathrm {T}}. \end{aligned}$$

The operator $$[\Delta _e]_{aIbJ}$$ applied to the nodal displacements of the interface element leads to the relative opening displacement between the $$(+)$$ and the $$(-)$$ interface flanks, i.e., $$[\Delta _e]_{aIbJ}U_{bJ}=[[U_{aI}]]$$$$(1\le a\le S_e,\, 1\le b\le 2S_e,\, 1\le I,J\le 3)$$.

Similarly, the operator $$[\Delta _e]_{ab}$$ applied to the nodal temperatures of the interface element leads to the temperature jumps between the $$(+)$$ and the $$(-)$$ interface flanks, i.e., $$[\Delta _e]_{ab} \varTheta _a=[[\varTheta _a]]$$$$(1\le a\le S_e,\, 1\le b\le 2S_e)$$. Analogous expressions hold for the test functions $$\delta U_K , [[ \delta U_K ]] $$, $$\delta \varTheta $$, and $$[[ \delta \varTheta ]]$$.

After introducing these expressions in the weak form (17), its discretized version reads:

21 $$\begin{aligned} \begin{aligned}&\sum _e \int _{R_e} \rho \varPhi _b \varPhi _a \dfrac{\partial ^2 U_{bI} }{\partial t^2} {\delta U}_{aI} \mathrm {d}V \\&\qquad +\sum _e \int _{R_e} C_{IJKL} \dfrac{\partial \varPhi _b}{\partial x_L} \dfrac{\partial \varPhi _a}{\partial x_J} U_{bK} {\delta U}_{aI} \mathrm {d}V \\&\qquad -\sum _e \int _{R_e} \varPhi _a \beta \delta _{KL} \dfrac{ \partial \varPhi _b }{\partial x_L } \varTheta _a {\delta U}_{bK} \mathrm {d}V\\&\quad =\sum _e \int _{S_e} t_J\varPsi _a \Delta _{aJbK} {\delta U}_{bK} \mathrm {d}A\\&\qquad + \sum _e \int _{\partial R_e} t^*_{I} \varPhi _a {\delta U}_{aI}\mathrm {d}A \end{aligned} \end{aligned}$$

Similarly, the discretized weak form (18) is:

22 $$\begin{aligned} \begin{aligned}&\sum _e \int _{R_e} \rho c \varPhi _a \varPhi _b \dfrac{ \partial \varTheta _a}{\partial t} \delta \varTheta _b \mathrm {d}V \\&\quad +\sum _e \int _{R_e} k \dfrac{\partial \varPhi _a }{\partial x_L } \dfrac{\partial \varPhi _b }{\partial x_L } \varTheta _a \delta \varTheta _b \mathrm {d}V\\&\quad +\sum _e \int _{\partial R_e} q^* \varPhi _a \delta \varTheta _a \mathrm {d}A \\&\quad +\sum _e \int _{R_e} T_0 \beta \delta _{KL} \dfrac{ \partial \varPhi _b}{\partial x_L } \dfrac{ \partial U_{bK} }{ \partial t} \varPhi _a \delta \varTheta _a \mathrm {d}V \\&\quad + \sum _e \int _{S_e} q \varPsi _a \Delta _{ac} \delta \varTheta _c \mathrm {d}A=0 \end{aligned} \end{aligned}$$

In matrix form, the previous discretized weak forms become:

23 $$\begin{aligned} \begin{aligned}&\sum _e \{ \delta U_e \}^T \left[ M^{uu}_e\right] \dfrac{ D^2 \{ U_e \} }{ D t^2 } + \sum _e \{ \delta U_e \}^T \left[ K^{u \theta }_e\right] \{ U_e \} \\&\quad +\sum _e \{ \delta U_e \}^T \left[ C^{u \theta }_e\right] \{ \varTheta _e \}= \sum _e \{ \delta U_e \}^T \{ F^u_e \} \\&\quad +\sum _e \{ \delta U_e \}^T \{ f^u_e \} \end{aligned} \end{aligned}$$

24 $$\begin{aligned} \begin{aligned}&\sum _e \{ \delta \varTheta _e \}^T [K^{\theta \theta }_e] \{ \varTheta _e \} + \sum _e \{ \delta \varTheta _e \}^T \left[ C^{u \theta }_e\right] \dfrac{ D \{ \varTheta _e \} }{ D t } \\&\quad + \sum _e \{ \delta \varTheta _e \}^T \left[ C^{u \theta }_e\right] \dfrac{ D \{ U_e \}}{D t} + \sum _e \{ \delta \varTheta _e \}^T \left\{ F^{\theta }_e \right\} \\&\quad +\sum _e \{\delta \varTheta _e \}^T \left\{ f^{\theta }_e \right\} =0 \end{aligned} \end{aligned}$$

where $\{U_e\}=(U_{11},U_{12},U_{13},\dots ,U_{1N},U_{1N},U_{1N})^{\mathrm {T}}$, $\{ \varTheta _e \} =(\varTheta _1,\dots ,\varTheta _N)^{\mathrm {T}}$, and *N* stands either for *N*(*e*) for a bulk element, or for 2*S*(*e*) for an interface element. Expressions for the matrix operators are detailed in Appendix.

By introducing the generalized displacement vector at the element level with the following ordering, $$\{ \Delta _e \}=(U_{11},U_{12},U_{13},\varTheta _1,\dots ,U_{N1},U_{N2},U_{N3},\varTheta _N)^{\mathrm {T}}$$$$=[P]^{\mathrm {T}} ( \{ U_e \},\{ \varTheta _e \})^{\mathrm {T}}$$, where *N* stands for either *N*(*e*) for the bulk elements, or 2 *S*(*e*) for the interface elements, Eqs. (23) and (24) combine as:

25 $$\begin{aligned} \begin{aligned}&\sum _e \{ \delta \Delta _e \}^T [M_e] \dfrac{ D^2 \{ \Delta _e \} }{ D t^2 } + \sum _e \{ \delta \Delta _e \}^T [C_e] \dfrac{ D \{ \Delta _e \} }{ D t } \\&\quad +\sum _e \{ \delta \Delta _e \}^T [K_e] \{ \Delta _e \}= \sum _e \{ \delta \Delta _e \}^T \{ F_e \}\\&\quad + \sum _e \{ \delta \Delta _e \}^T \{ f_e \} \end{aligned} \end{aligned}$$

where the expressions for the mass, $[M_e]$, the damping, $[C_e]$, and the stiffness matrix, $[K_e]$, as well as for the load vector, $\{F_e\}$, and the interface load vector, $\{f_e\}$, are collected in the Appendix. Note that to pass from Eqs. (23) and (24) to Eq. (25), a permutation matrix [*P*] has been used, see again Appendix for more details.

Let $\{ \Delta \}$ be the global displacement vector and $[L_e]$ the localization matrix that selects the element nodal values, viz.:

26 $$\begin{aligned} \{ \Delta _e \}=[L_e] \{ \Delta \} \end{aligned}$$

hence, Eq. (25) can be recast as:

27 $$\begin{aligned} \begin{aligned}&\{ \delta \Delta \}^T [M] \dfrac{ D^2 \{ \Delta \} }{ D t^2 } + \{ \delta \Delta \}^T [C] \dfrac{ D \{ \Delta \} }{ D t }\\&\quad +\{ \delta \Delta \}^T [K] \{ \Delta \}= \{ \delta \Delta \}^T \{ F \} + \{ \delta \Delta \}^T \{ f \} \end{aligned} \end{aligned}$$

where the global mass, damping and stiffness matrices and the load vector are assembled as follows:

$$\begin{aligned}{}[M]&=\sum _e [L_e]^T [M_e] [L_e] \ , \{ F \} = \sum _e [L_e]^T \{ F_e \} , \\ [K]&=\sum _e [L_e]^T [K_e] [L_e] \ ,[C]=\sum _e [L_e]^T [C_e] [L_e] , \\ \{ f \}&= \sum _e [L_e]^T \{ f_e\} \end{aligned}$$

By simplifying the virtual variation of the test function $\{ \delta \Delta \}$ and neglecting the inertial term, the thermo-mechanical problem requires the solution of the following nonlinear set of equations:

28 $$\begin{aligned}{}[C] \dfrac{ D}{ D t} \{ \Delta \} + [K] \{ \Delta \}= \{ F \} + \{ f \} \end{aligned}$$

### Discretized weak form of moisture diffusion

The discretization of the weak form for moisture diffusion is derived by introducing a finite element mesh of the mid-surface *S*(*p*) of the encapsulant layer. In principle, since a staggered geometrical multiscale solution scheme is adopted, the spacing of the mesh used to solve moisture diffusion can be different from that used for the discretization of the thermo-mechanical problem. In that case, a projection of the nodal temperatures from the discretized thermo-mechanical problem to the nodes of the mesh used to solve moisture diffusion has to be performed via a suitable interpolation scheme. In the sequel, without any loss of generality, we consider a finite element discretization for moisture diffusion coincident with the middle surface discretization of each interface element, i.e., $S(p)\approx \bigcup _e S_e$.

By introducing the shape functions $\varPsi _a$, the water concentration in a generic point of coordinates $(x_1,x_2)$ and at a time *t* is

29 $$\begin{aligned} C(x_1,x_2,t)=\sum ^{S(e)}_{a=1} \varPsi _a(\xi _1 , \xi _2) C_a(t) \end{aligned}$$

Introducing Eq. (29) into (19), we obtain:

30 $$\begin{aligned} \sum _e \int _{S_e} D C_a \dfrac{ \partial \varPsi _a }{ \partial x_I } \dfrac{ \partial \varPsi _b }{ \partial x_I } \delta C_b \mathrm {d}A\,+\sum _e \int _{S_e} \dfrac{ \partial C_a }{ \partial t} \varPsi _a \varPsi _b \delta C_b \mathrm {d}A=0 \end{aligned}$$

providing the following matrix form:

31 $$\begin{aligned} \sum _e \{ \delta C \}^T [B_e] \{ C_e \} + \sum _e \{ \delta C_e \}^T [A_e] \dfrac{ D }{D t} \{ C_e \} =0 \end{aligned}$$

where the expression for the matrices $[A_e]$ and $[B_e]$ is detailed in the Appendix, and $\{ C_e \}=(C_1,\dots C_{S(e)})^{\mathrm {T}}$ is the vector collecting the nodal concentrations.

Introducing as previously the global node vector $\{ C \}$ of unknowns and the localization matrix $[L_e]$ such that:

32 $$\begin{aligned} \{ C_e \}=[L_e] \{ C \} \end{aligned}$$

the assembling of the global matrices leads to:

$$\begin{aligned}{}[B]= \sum _e [L_e]^T [D_e] [L_e],\quad [A]= \sum _e [L_e]^T [A_e] [L_e] \end{aligned}$$

providing a linear system of ordinary differential equations:

33 $$\begin{aligned}{}[A] \dfrac{D}{D t} \{ C \} + [B(t)] \{ C \}= \{ 0 \} \end{aligned}$$

Note that the matrix [*B*] is time dependent because it contains the diffusivity *D* which changes with time according to (16).

## Proposed numerical solution scheme

To solve numerically the system (28) resulting from the thermo-mechanical problem, we adopt an Euler backward implicit scheme so that:

34 $$\begin{aligned} \dfrac{D}{D t} \{ \Delta \}^{n+1} \approx \dfrac{1}{ \Delta t} \left( \{ \Delta \}^{n+1} - \{ \Delta \}^{n} \right) \end{aligned}$$

where $n=1,2, \dots ,N$ and $t^n=t^{n-1}+ \Delta t$. Hence, Eq. (28) becomes:

35 $$\begin{aligned} \dfrac{1}{\Delta t} [D] \left( \{ \Delta \}^{n+1} - \{ \Delta \}^{n} \right) + [K] \{ \Delta \}^{n+1}= \{ F \}^{n+1} \end{aligned}$$

which is a nonlinear system of equations in the unknown $\{ \Delta \}^{n+1}$, where the nonlinearity relies in the load vector due to the nonlinear relations between the cohesive tractions and the relative displacements, and between the heat flux and the temperature jump at the polymeric interfaces.

This problem is solved iteratively using a Newton-Raphson scheme. At the iteration $k+1$ we have an approximation $\{ \Delta \}^{n+1}_{(k)}$ for $\{ \Delta \}^{n+1}$ and we seek for a better approximation $\{ \Delta \}^{n+1}_{(k+1)}$ such that:

36 $$\begin{aligned} \{ \Delta \}^{n+1}_{(k+1)}= \{ \Delta \}^{n+1}_{(k)} + \{ d \Delta \}^{n+1}_{(k)} \end{aligned}$$

By introducing $\{ \Delta \}^{n+1}_{(k)}$ into $\{ F \}^{n+1}$, we obtain $\{ F \}^{n+1}_{(k)}$. Linearization of $\{F\}^{n+1}_{(k+1)}$ leads to

37 $$\begin{aligned} \{ F \}^{n+1}_{(k+1)}= \{ F \}^{n+1}_{(k)} + \left[ \dfrac{ \partial \{ F \} }{ \partial \{ \Delta \} } \right] ^{ n+1 }_{(k)} \{ d \Delta \}^{n+1}_{(k)} \end{aligned}$$

By substituting this expression back to the system (35) and rearranging the various terms, leads:

38 $$\begin{aligned}&\left( \dfrac{1}{\Delta t} [C]+ [K] - \left[ \dfrac{ \partial \{ F \} }{ \partial \{ \Delta \} } \right] ^{ n+1 }_{(k)} \right) \{ d \Delta \}^{n+1}_{(k)}=\end{aligned}$$

39 $$\begin{aligned}&\quad - \dfrac{1}{ \Delta t} [C] \left( \{ \Delta \}^{n+1}_{(k)} - \{ \Delta \}^{n} \right) - [K] \{ \Delta \}^{n+1}_{(k)} + \{ F \}^{n+1}_{(k)} \end{aligned}$$

where the right-hand side is the so-called residual vector, $\{ R \}^{n+1}_{(k)}$, so that we can write:

40 $$\begin{aligned} \left( \dfrac{1}{\Delta t} [C]+ [K] - [T]^{n+1}_{(k)} \right) \{ d \Delta \}^{n+1}_{(k)}=\{ R \}^{n+1}_{(k)} \end{aligned}$$

where:

41 $$\begin{aligned} \begin{aligned}&[T]^{n+1}_{(k)} = \left[ \dfrac{ \partial \{ F \} }{ \partial \{ \Delta \} } \right] ^{ n+1 }_{(k)}= \sum _e [L_e]^T \left[ \dfrac{ \partial \{ F_e \} }{ \partial \{ \Delta _e \} } \right] ^{ n+1 }_{(k)}[L_e] \\&\quad = \sum _e [L_e]^T \left[ T_e \right] ^{n+1}_{(k)} [L_e] \end{aligned} \end{aligned}$$

and $[T_e]^{n+1}_{(k)}$ is given by

42 $$\begin{aligned} \begin{aligned}&[T_e]^{n+1}_{(k)}= [P]^T \begin{bmatrix} \left[ T^{uu}_e \right]&\quad \left[ T^{u \theta }_e \right] \\ \left[ T^{\theta u}_e \right]&\quad \left[ T^{\theta \theta }_e \right] \end{bmatrix}^{n+1}_{(k)} [P]\\&\quad = [P]^T \begin{bmatrix} \left[ \dfrac{\partial \{ f^u_e \}}{ \partial \{ U_e \} } \right]&\quad \left[ \dfrac{\partial \{ f^u_e \}}{ \partial \{ \varTheta _e \} } \right] \\ \left[ \dfrac{\partial \{ f^{\theta }_e \}}{ \partial \{ U_e\} } \right]&\quad \left[ \dfrac{\partial \{ f^{\theta }_e \}}{ \partial \{ \varTheta _e \}} \right] \end{bmatrix}^{n+1}_{(k)} [P] \end{aligned} \end{aligned}$$

The various terms entering Eq. (42) are reported in the Appendix.

Once temperature and displacements are computed at a given time step $n+1$, these nodal results are transferred to the discretized moisture diffusion problem. Its solution is then performed by using an Euler backward time integration scheme with the same partition of the temporal interval $[0,t_f]$ as for the thermo-mechanical problem. For $n=1, \dots , N$ time steps $\Delta t$, we solve the linear system of equations:

43 $$\begin{aligned} \left( \Delta t [B]^{n+1}+ [A] \right) \{ C \}^{n+1}=[A] \{ C \}^n \end{aligned}$$

where

44 $$\begin{aligned}{}[B_e]^{n+1}_{ab}=\int _{S_e} D(\left\langle \varTheta \right\rangle ^{n+1}, [[ U_3 ]]^{n+1}) \dfrac{ \partial \varPsi _a }{ \partial x_I } \dfrac{ \partial \varPsi _b }{ \partial x_I } \mathrm {d}A \end{aligned}$$

are computed using the values $\left\langle \varTheta \right\rangle ^{n+1}, [[ U_3 ]]^{n+1}$ obtained from the thermo-mechanical problem.

The algorithm for the proposed time stepping method with a staggered scheme is detailed in Algorithm 1. The Newton-Raphson iteration is performed until machine precision is achieved, i.e., up to a tolerance in the norm of the residual vector $\mathrm {tol}=1\times 10^{-15}$.

It has to be remarked that the time-dependency of the visco-elastic constitutive equation (10) requires the use of a history variable $\{h_v\}$ for all the nodes of the finite element mesh for the thermo-mechanical problem. To model relaxation, this variable is set to zero at any change of temperature (state variable), while it is updated by the current time increment if the temperature remains constant with respect to the previous time step, within a given tolerance $\text {tol}_2$. This method allows the simulation of the thermo-viscoelastic behavior of polymeric materials when the temperature-time superposition principle does not apply, for instance due to a change of microstructure by varying temperature as it happens in the case of epoxy-vinil-acetate used in photovoltaics.
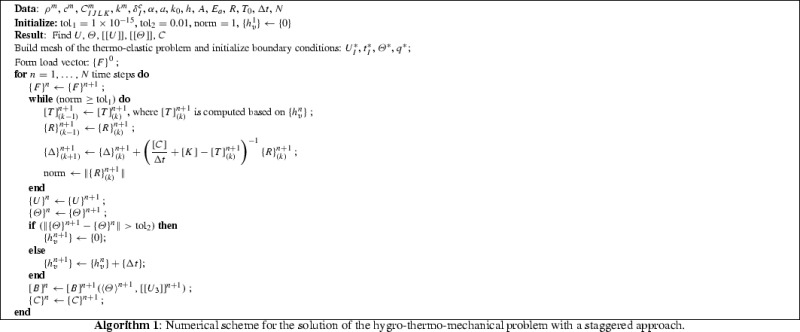


## Application to photovoltaics

In this section we propose the simulation of the two tests prescribed by international standars [4], namely the *damp heat* test and the *humidity freeze* test. While the former allows the complete uncoupling between the solution of the thermo-mechanical problem from moisture diffusion and allows the derivation of a closed form solution useful for benchmarking, the latter requires the present fully-coupled solution scheme. Moreover, the role of a temperature-dependent diffusion coefficient and the role of cohesive cracks in Silicon are investigated, in comparison to experimental results.

### Damp heat test

**Fig. 6:**
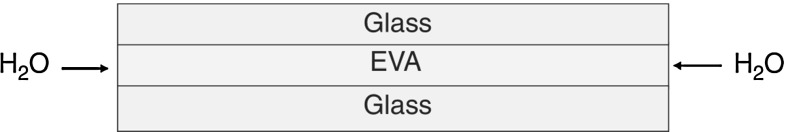
Sketch of the *damp heat* test

Let us consider a laminate of span $L=125$ cm and made of a Glass-Glass structure separated by EVA as in Fig. 6. The thickness of each glass is 3 mm, while the thickness of the EVA is 0.5 mm. In this laminate, moisture is diffusing from the free edges towards the centre, since glass is not permeable to moisture.

As far as the initial and boundary conditions are concerned, let us consider the prescriptions by international standards [4] for the *damp heat* test, that is, a constant temperature of $85\,^{\circ }$C and an air relative humidity of $85\%$. This relative humidity corresponds to a moisture content $c^*=0.55$ g/cm$^3$ imposed at the free edges of the laminate, i.e., for $x_1=0$ and $x_1=L$, where $x_1$ is the distance from the left edge of the PV module.

Since temperature is held constant, the problem of diffusion can be solved independently from the thermo-mechanical problem, considering a constant diffusivity $D=5 \times 10^{-5}\mathrm {cm}^2/\mathrm {s}$ corresponding to $85\,^{\circ }$C. For this special case, the analytical solution was obtained in [12] and it is used as a benchmark for our computational scheme:

45 $$\begin{aligned} \begin{aligned} c(x_1,t)&=c^* - \dfrac{4 c^*}{ \pi } \sum ^{\infty }_{k=0} \dfrac{1}{(2k+1)} \sin \left( \dfrac{(2k+1) \pi x_1}{L} \right) \\&\quad \times \mathrm {exp} \left( - \dfrac{(2k+1)^2 \pi ^2 D t}{L^2} \right) \end{aligned} \end{aligned}$$

**Fig. 7:**
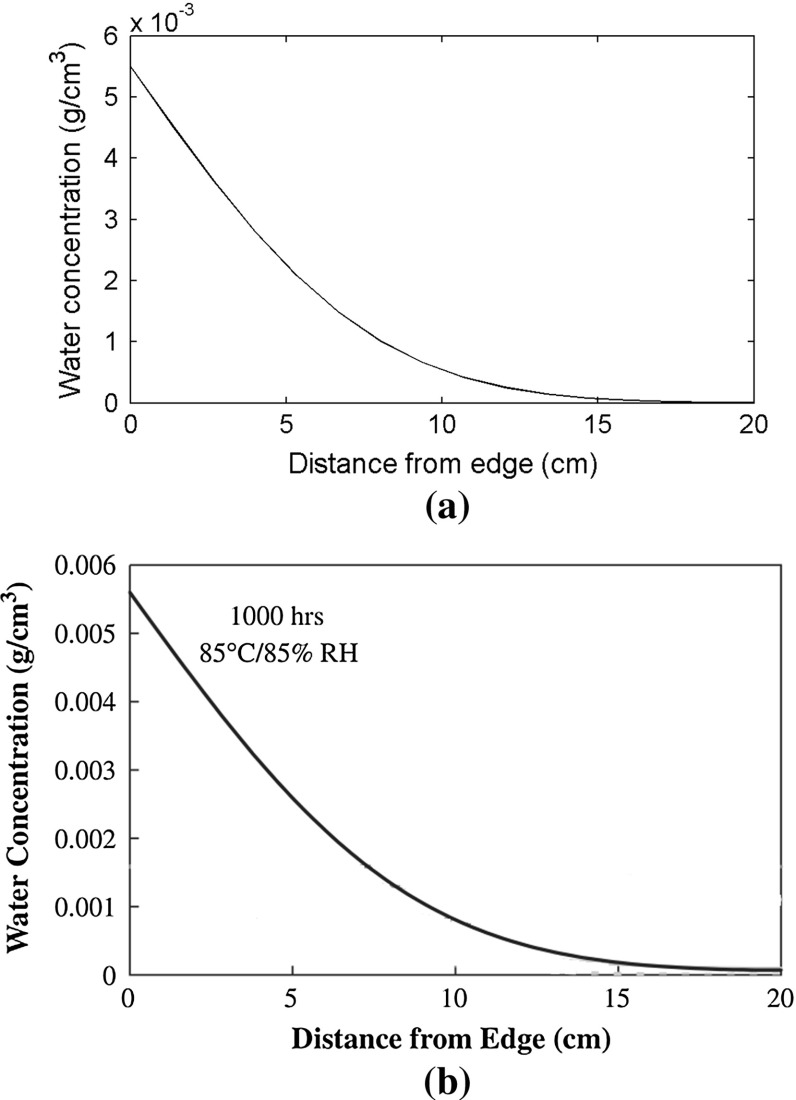
Moisture concentration in the encapsulant after 1000 h. **a** Numerical simulation; **b** analytical solution by [12]

After 1000 h, the predicted moisture concentration is shown in Fig. 7a and its distribution in the EVA layer matches exactly the reference one in Fig. 7b.

### Humidity freeze test

**Fig. 8:**
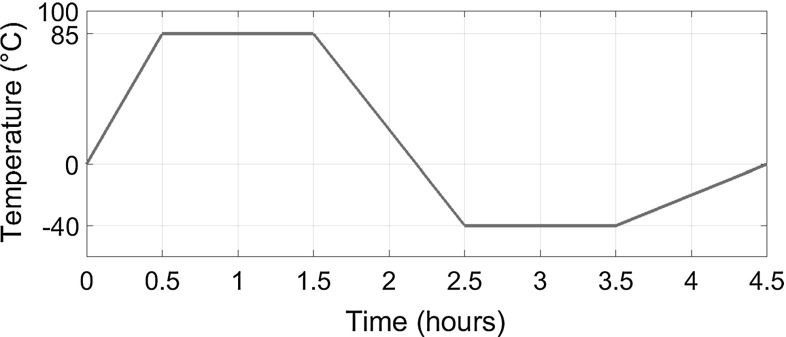
Temperature profile of $\theta ^*$ imposed inside the climate chamber during the *humidity freeze* test

In this test requested by international standards [4], PV modules simply supported along their edges are subjected to a cycling temperature from $-40$ up to $85\,^{\circ }$C with the following ramps (see also the sketch in Fig. 8):

$$\begin{aligned} \theta ^*(t)={\left\{ \begin{array}{ll} \dfrac{\theta ^*_1}{t^*_1} t \ \qquad \qquad \qquad \qquad \qquad \quad 0 \le t < t^*_1 \\ \theta ^*_1 \ \ \qquad \qquad \qquad \qquad \qquad \quad t^*_1 \le t < t^*_2 \\ \theta ^*_2 - \dfrac{\theta ^*_1 - \theta ^*_2}{t^*_3-t^*_2 }(t^*_3-t) \ \quad \ t^*_2 \le t < t^*_3 \\ \theta ^*_2 \ \ \qquad \qquad \qquad \qquad \qquad \quad t^*_3 \le t < t^*_4 \\ \dfrac{ \theta ^*_2 }{t^*_5-t^*_4}(t^*_5-t) \ \ \qquad \quad \ \ \quad t^*_4 \le t < t^*_5 \\ \end{array}\right. } \end{aligned}$$

where $$\theta ^*_1=$$$$85^{\circ }$$C, $$\theta ^*_2= $$$$-40^{\circ }$$C, and $$t^*_1=0.5$$ h, $$t^*_2=1.5$$ h, $$t^*_3=2.5$$ h, $$t^*_4=3.5$$ h, $$t^*_5=4.5$$ h.

The relative humidity in the air is kept constant at $85\%$ for the range of temperatures where its control is thermodynamically feasible.

**Fig. 9:**
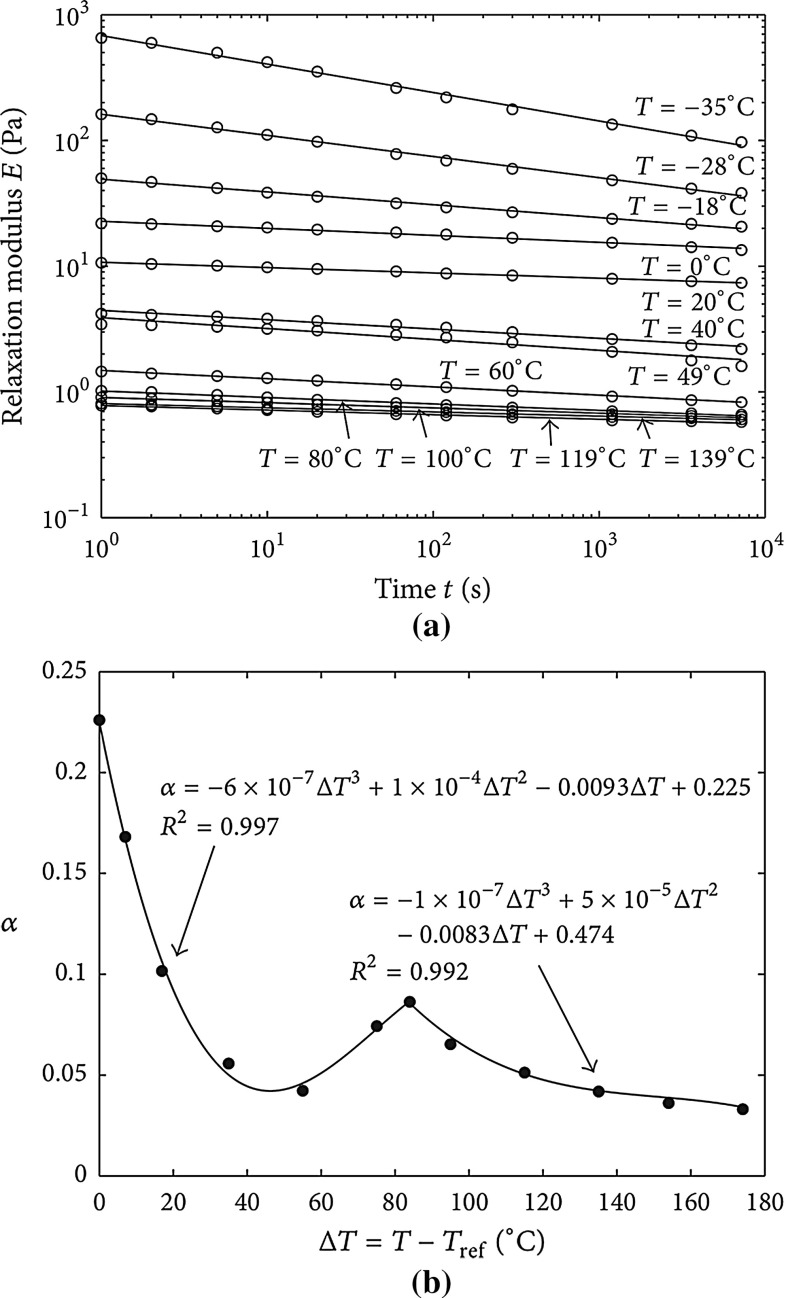
**a** EVA relaxation modulus versus time at various temperatures in a double logarithmic scale; **b** temperature dependent fractional exponent $\alpha (T)$, adapted from [21]

Due to a non constant temperature boundary condition, this problem is much more difficult to be simulated as compared to the *damp heat* test. In particular, the cohesive properties of EVA have to be updated during the simulation, as well as its diffusivity. More specifically, as far as the Young’s modulus of EVA is concerned, the parameters $\alpha (T)$ and *a*(*T*) are herein considered to be temperature-dependent as experimentally evaluated in [6] and interpreted via a fractional calculus model in [21], see the plot for $\alpha (T)$ and *E*(*T*) in Fig. 9.

**Fig. 10:**
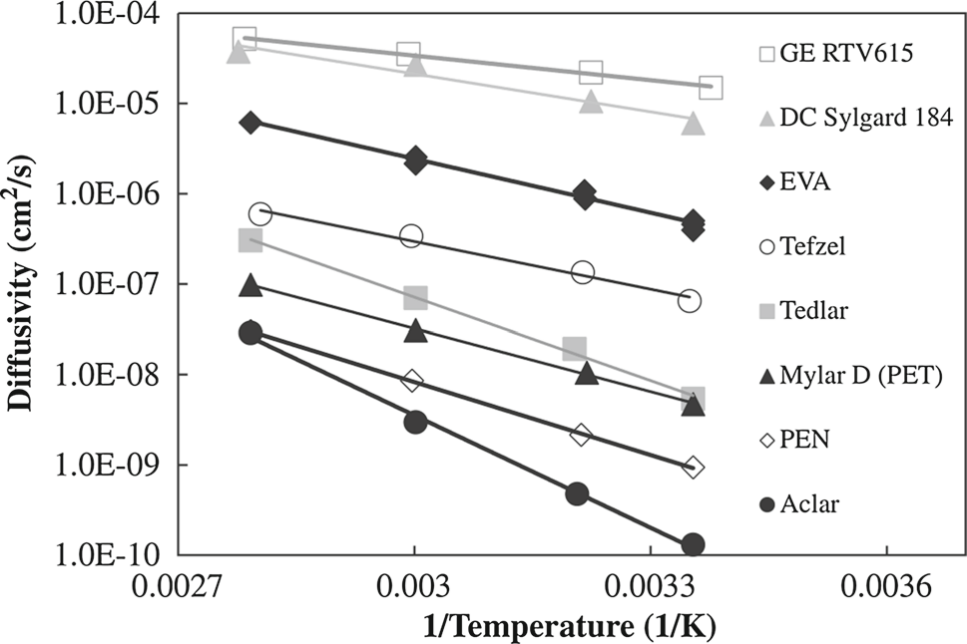
Diffusivity of various encapsulant materials versus the inverse of temperature, adapted from [12]

Regarding the diffusive properties, we consider the expression of *D*(*T*) for EVA as reported in [12] and shown in Fig. 10. Such trends can be fitted according to the Arrhenius type equation (16).

The critical crack opening, $\delta _3$, to be assigned to the cohesive zone model can be estimated from published experimental data in [13] reporting the variation of the Mode I fracture energy with temperature. Since the fracture energy *G* is the area below the traction-separation curve, the following relation holds:

$$\begin{aligned} G=\int ^{\delta ^c_3}_0 t_3([[ u_3 ]], \left\langle \theta \right\rangle ) d[[ u_3 ]]= \dfrac{1}{2}\dfrac{\left( \delta ^c_3 \right) ^2}{h_{\mathrm {EVA}}} E_{\mathrm {EVA}}(t, T) \end{aligned}$$

**Fig. 11:**
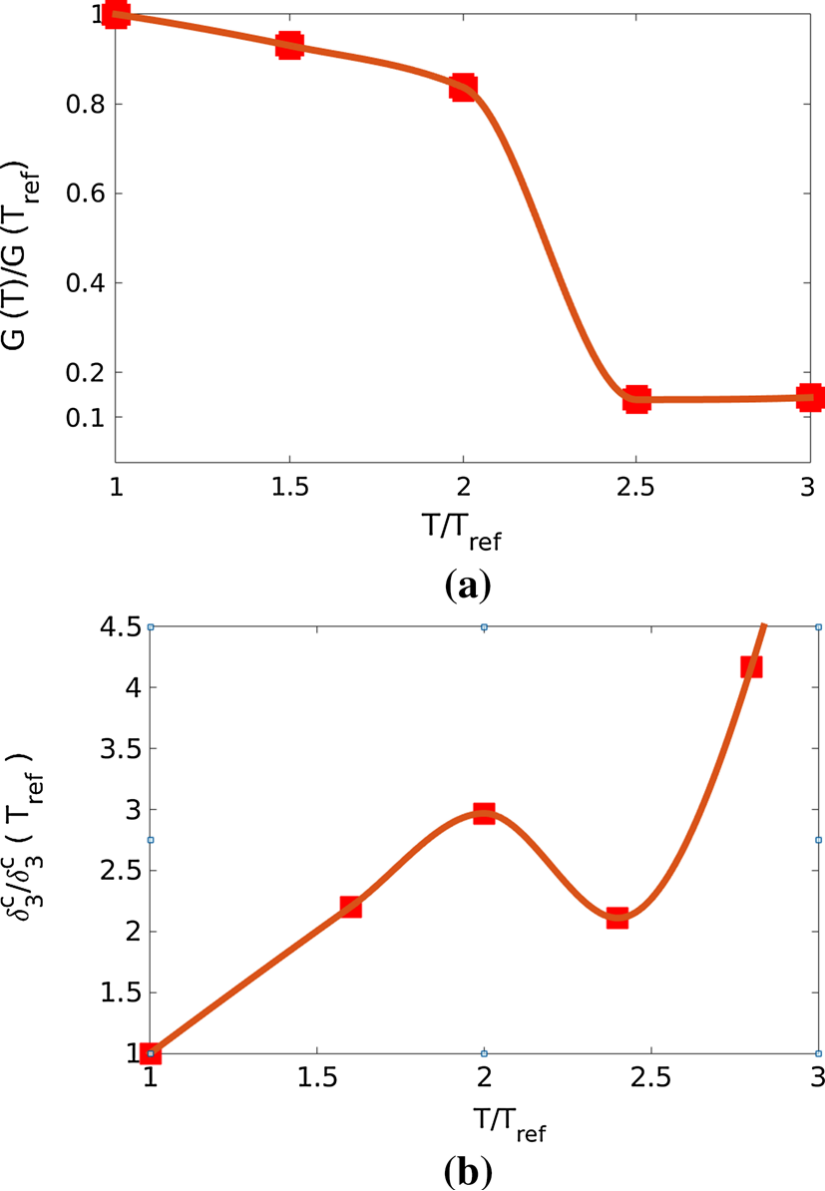
**a** EVA cohesive energy from [13]; **b** critical gap opening versus temperature ($T_{\mathrm {ref}}=25\,^{\circ }$C)

Hence, *G*(*T*) experimental data can be converted in $\delta _3^c(T)$ data based on the known temperature dependency of the Young’s modulus of EVA as in Fig. 8a, evaluated for the asymptotic condition of an infinite time. Based on these data, we obtain the correlations shown in Fig. 11 and used as input for the numerical simulations.

**Fig. 12:**
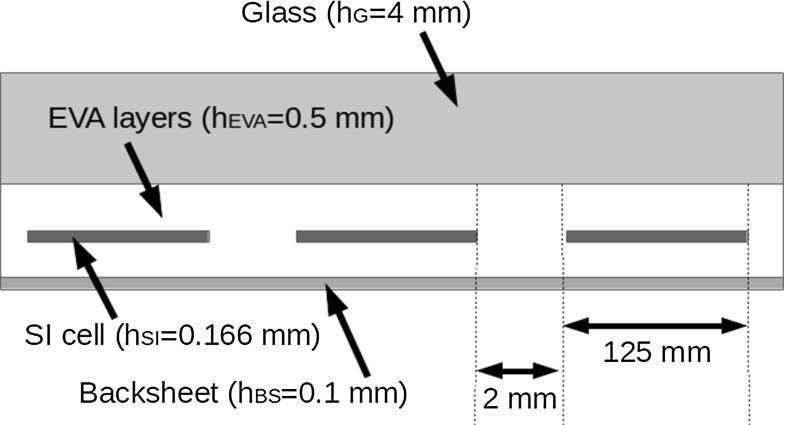
Sketch of the cross-section of a PV mini-module used in the simulation (not in scale)

A sketch of the cross-section of a PV mini-module simulated in the present study and containing 3 solar cells is shown in Fig. 10. The lateral size of each Silicon cell is 125 mm and the the interspace between two cells is 2 mm. The module is made of a glass superstrate with thickness $h_{G}=4$ mm, an encapsulating polymer layer (EVA) with thickness $h_{\mathrm {EVA}}=0.5$ mm, the Silicon solar cell with thickness $h_{\mathrm {SI}}=0.166$ mm, another layer of EVA with the same thickness as the previous one, and finally a thin backsheet made of an ethylene tetrafluoroethylene core with silicon nitride coating (isovolta Icosolar T 2754), with thickness $h_{\mathrm {BS}}=0.1$ mm. Thermal and mechanical parameters of each layer are collected in Table 1 and are taken from [5, 6]. Since moisture penetrates from the backsheet and percolates along the interspace between solar cells, in the numerical simulations it is possible to impose a constant value of moisture concentration, $c^*$, directly at the boundary of each solar cell embedded in the PV module (Fig. 12).

**Table 1:** Material parameters for the layers

	*E* (GPa)	$$\alpha $$	$$\rho $$ (Kg/$$m^3)$$	$$c_{\varepsilon }$$ (W/mK)	*k* (J/mKg)
Glass	73	$8e-6$	2300	500	0.8
Si	130	$2.49e-6$	2500	715	148
B.S.	2.8	$5.04e-5$	1000	300	0.36

**Fig. 13:**
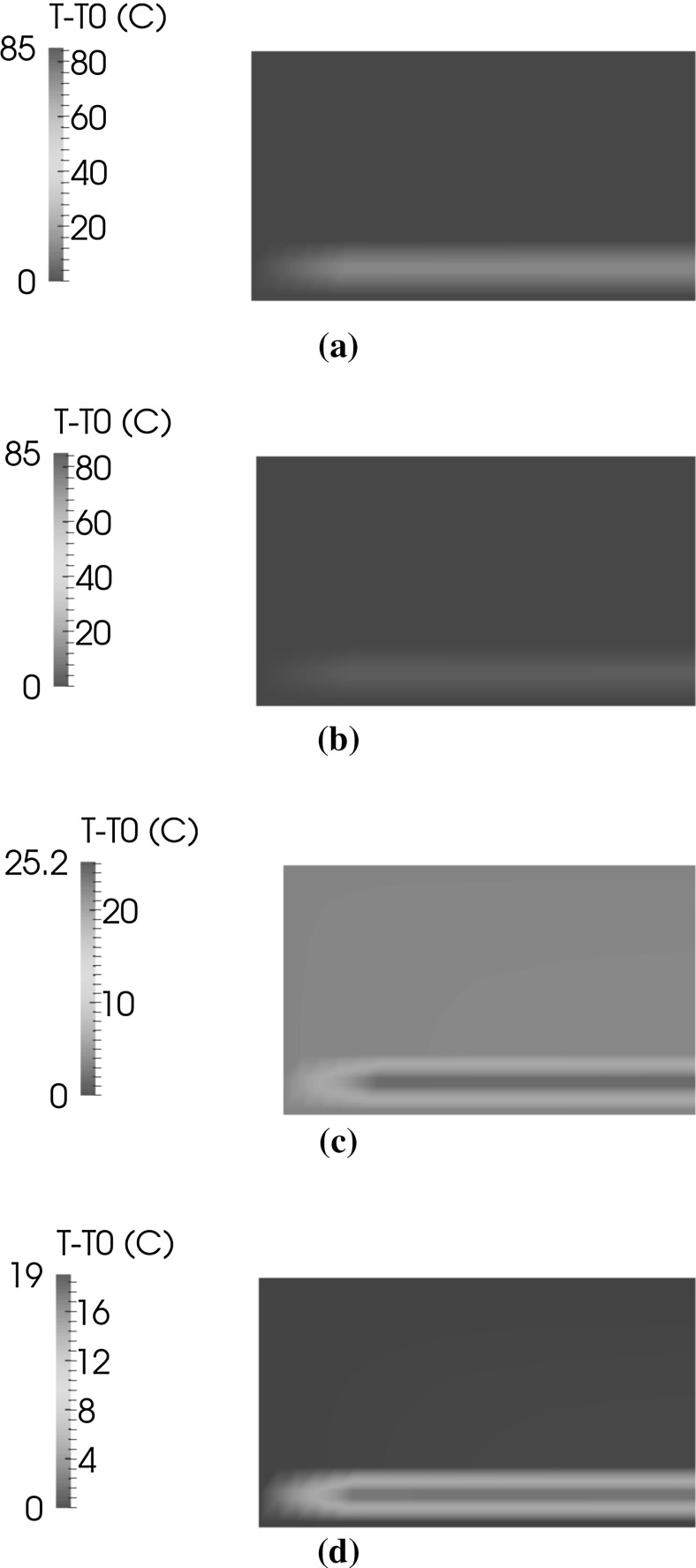
Contour plot of temperature inside the module for selected time steps **a** after 0.5 h, **b** after 1.5 h, **c** after 2 h, **d** after 2.25 h

**Fig. 14:**
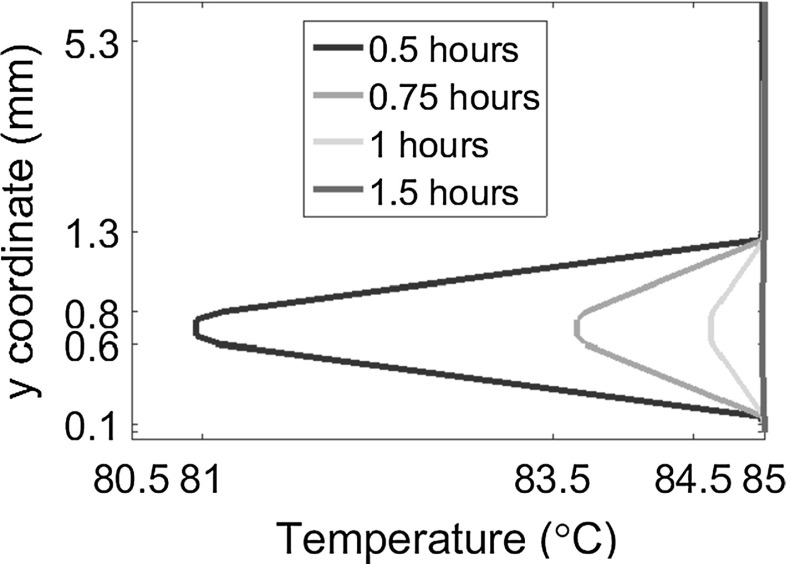
Evolution of temperature over a *vertical line* at $x_1=2$ mm from the lateral side, during the first ramp from 0 to $85\,^{\circ }$C

The temperature distribution inside a portion of the PV module cross-section near one of the free boundaries is shown in the contour plots in Fig. 13 for selected time steps. After the first ramp from 0 to $85\,^{\circ }$C completed after 0.5 hours, heat has diffused inside the panel and temperature is almost uniform everywhere and equal to $85\,^{\circ }$C. During the subsequent decreasing ramp from 85 to $-40\,^{\circ }$C, the Silicon cells and the EVA around them remain warmer than the other component. This temperature mismatch progressively shrinks during the further stage of the simulation at constant temperature $\theta ^*$. This trend is quantified in Fig. 14 by plotting the temperature along a vertical line at $x_1=2$ mm from the free edge of the laminate, through the panel thickness.

**Fig. 15:**
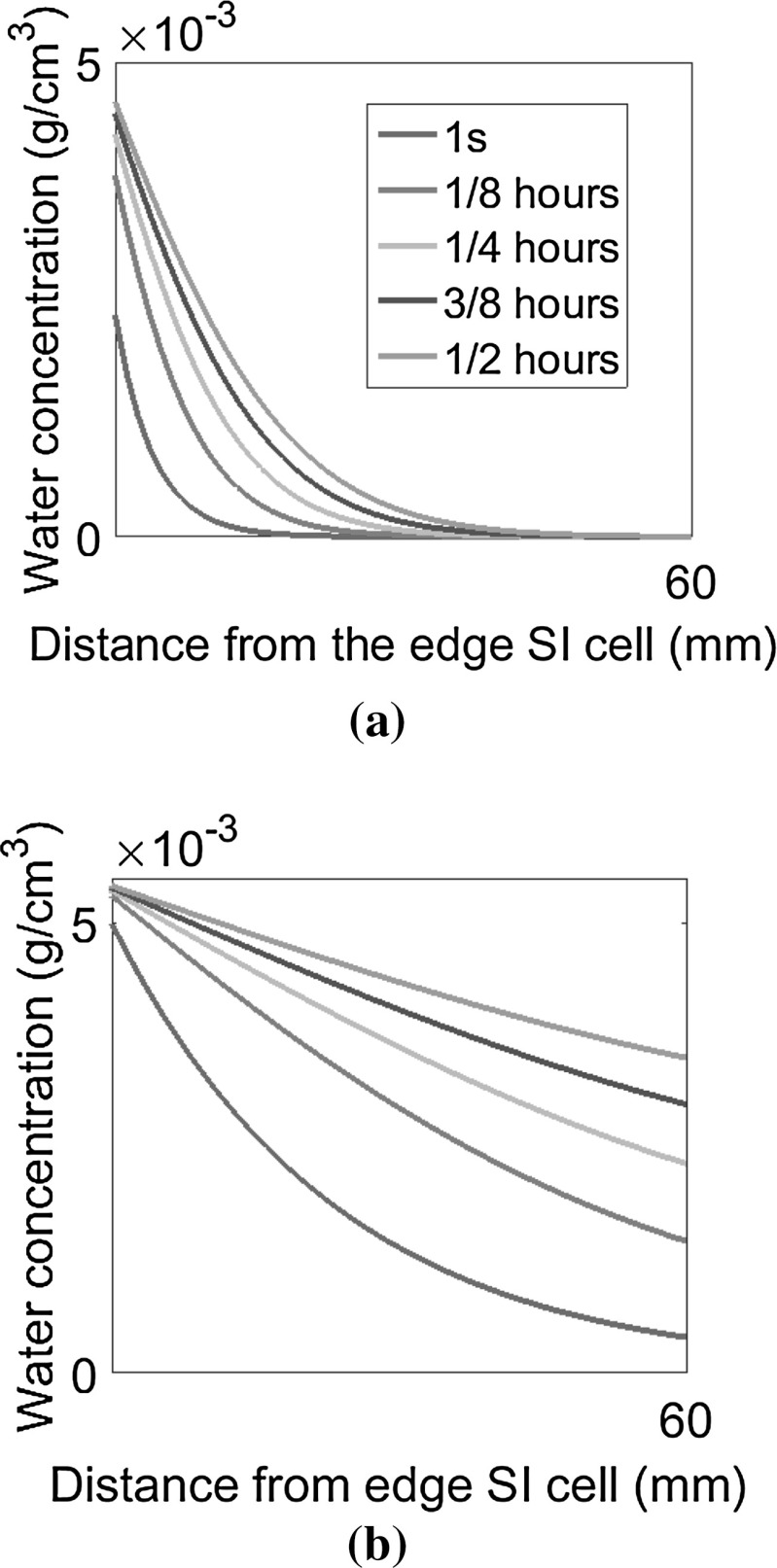
Evolution of moisture concentration in the EVA encapsulant for an updated or a constant diffusivity **a** *D* dependent on *T*, **b** $D = D$ ($85\,^{\circ }$C)

The evolution of moisture concentration in the encapsulant vs. time by using a time-dependent diffusivity is shown in Fig. 15a. The same simulation with a constant diffusivity $D=5 \times 10^{-5}$ cm$^2$/s corresponding to $85^{\circ }$C is shown for comparison in Fig. 15b. As it can be noticed, the proper update of the diffusivity based on the actual temperature of EVA during the simulation provides very different results from those based on a constant diffusivity. In particular, moisture diffusion is a much slower phenomenon than what expected by the approach presented in [12], which is based on the use of a constant diffusivity equal to that at the maximum temperature experienced during the test.

**Fig. 16:**
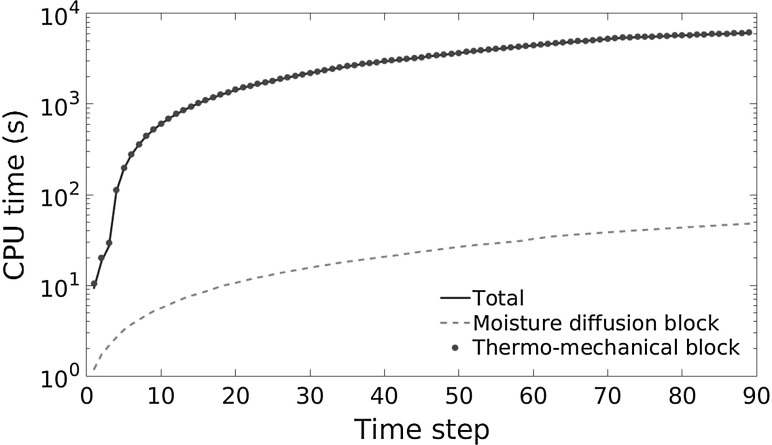
CPU time requested for the solution of the two blocks of the staggered scheme and for the total problem versus number of time steps

The time step used for the simulation of the *humidity freeze* test is $\Delta t=18$ s. At each time step, the Newton-Raphson iterative scheme used to solve the nonlinear thermo-mechanical block has a quadratic convergence and it requires a maximum of four iterations to satisfy the condition of a relative residual error norm less than the machine precision. For given variables computed from the solution of the thermo-mechanical block, the moisture diffusion block is linear and therefore it converges in one single iteration. In terms of CPU time, results are shown in Fig. 16, where the plot of the logarithm of CPU time for the solution of the nonlinear thermo-mechanical block, the moisture diffusion block, and the total time of the staggered scheme is shown for the first 90 time steps of the simulation of the *humidity freeze* test, corresponding to the first temperature ramp from 0 up to $85\,^\circ$C. Simulations have been run on the server Proliant DL585R07 (128 GB Ram, 4 processors AMD Opteron 6282 SE 2.60 GHz, 16 cores). In the case of delamination, which has not been observed in the simulation, the update of the EVA fracture energy based on the moisture content would require a further solution of the thermo-mechanical problem.

**Fig. 17:**
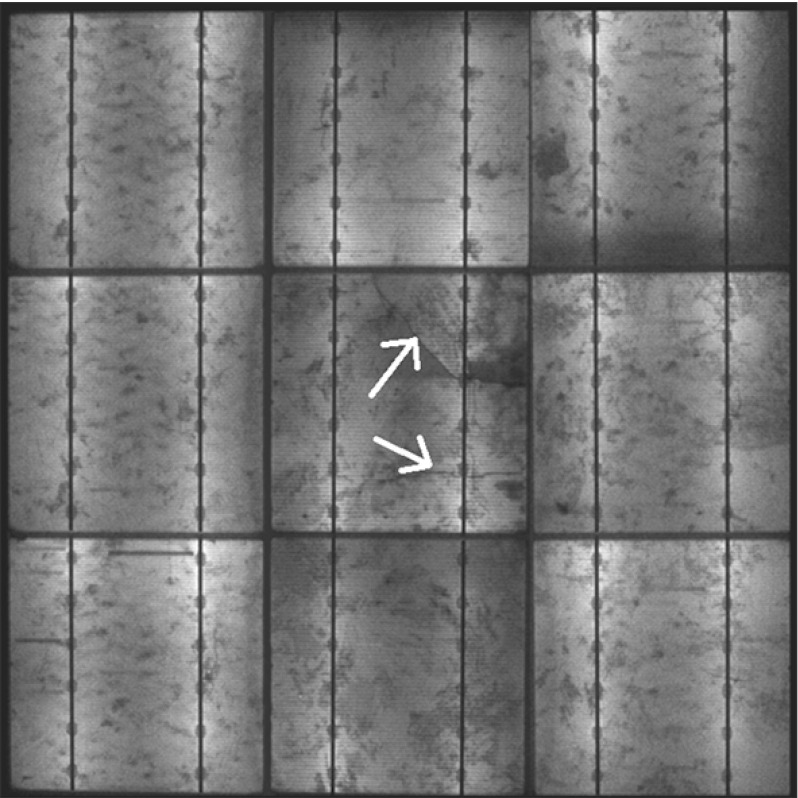
Electroluminescence image of the minimodule after three-point bending and before the *humidity freeze* test. Two main cracks in the central solar cell are shown by *arrows*

The proposed numerical method can be also employed to simulate the effect of cracks in Silicon on the evolution of moisture concentration. To have a benchmark experimental result to compare with, minimodules composed of $3\times 3$ multicrystalline solar cells with glass cover were realized in the Institute for Solar Energy Research of Hamelin, Germany (courtesy of Dr. M. Köentges) and were subjected to a three-point bending test to induce cracks in the central solar cell, see Fig. 17.

**Fig. 18:**
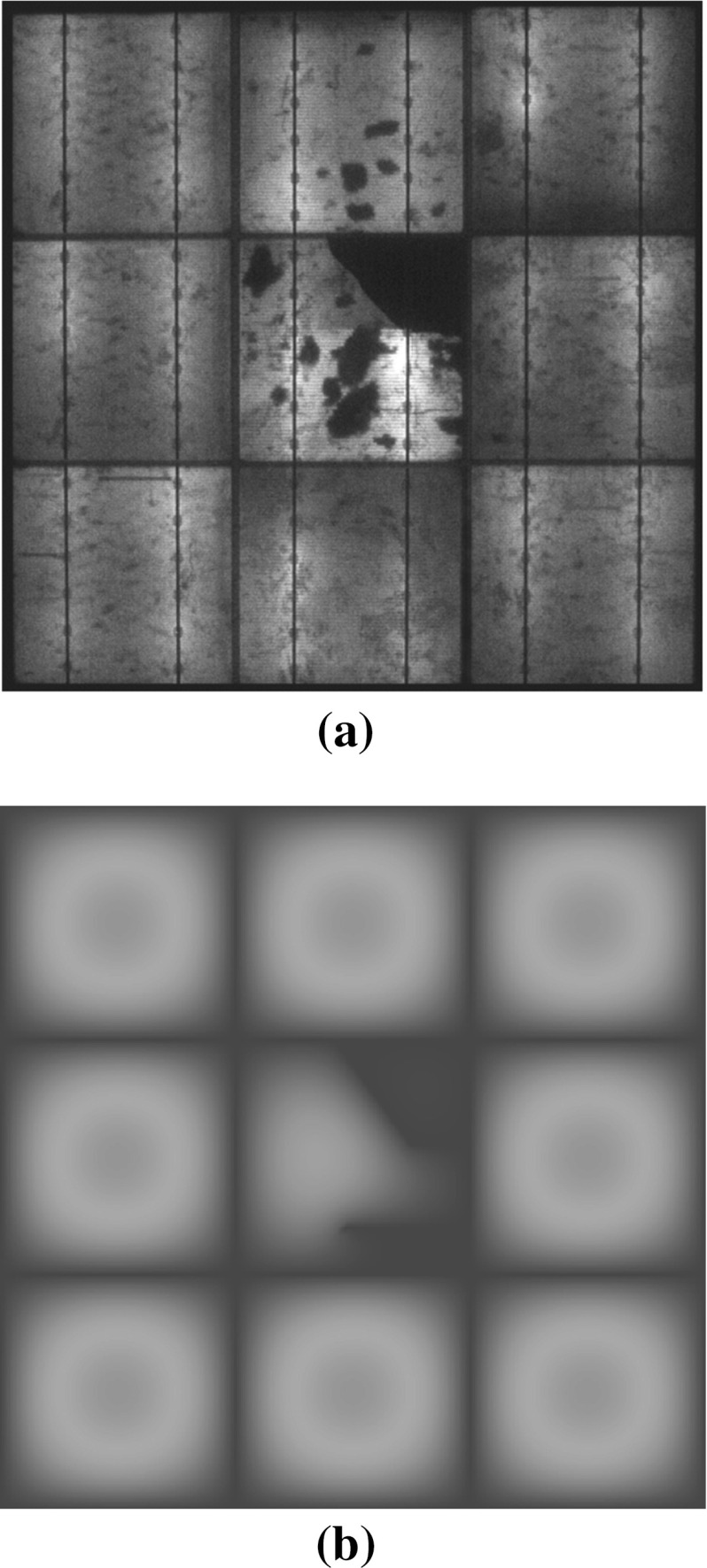
**a** Electroluminescence image showing electrically damaged areas in *black*; **b** Moisture concentration inside the EVA encapsulant. Experimental results and numerical predictions correspond to the scenario after 2400 h of *humidity freeze* test

This minimodule was then subjected to the *humidity freeze* test inside a climate chamber at Politecnico di Torino, Italy. Electroluminescence images taken regularly during the test (the reader is referred to [22] for more details about this nondestructive technique) show electrically damaged areas (black region) near the crack, see the EL image after 2400 h of testing in Fig. 18a. This electric degradation is presumably induced by chemical oxidation of the grid line deposited on the surface of the solar cell, enhanced by moisture. The presence of a crack appears to be relevant, since all the other solar cells show much lower degradation, mostly in form of dimmer areas at the edges of the solar cells due to moisture diffusion from the backsheet through the EVA interspace.

To prove that cracks can enhance electric degradation due to moisture diffusion through them, a numerical simulation is performed according to the present computational framework by imposing the value of $c^*=0.055$ g/cm$^3$ along the back side of the PV module. Diffusion can take place now not only along the EVA interspaces, but also along the two channel cracks, where 3D diffusive finite elements are inserted. Moisture concentration inside the EVA layer above the solar cells is shown in Fig. 18b after 2400 h of *humidity freeze* test. The presence of cracks enhances moisture diffusion in the central cell, with a contour plot of moisture concentration that correlates very well with the EL image of the electrically damaged areas (compare the red areas in Fig. 18b having $c=c^*$ with the black areas in Fig. 18a, confirming that electric degradation can be significantly enhanced by the presence of cracks.

## Conclusions and outlook

A comprehensive finite element computational framework for the simulation of coupled hygro-thermo-mechanical problems in photovoltaic laminates has been proposed. To achieve the computational efficiency required to simulate large scale commercial PV modules consisting of up to 60 solar cells, the EVA encapsulant layers have been modelled as zero-thickness interface elements whose traction-separation relations take into account the complex thermo-visco-elastic rheological response of the polymer as per experiments. Moreover, a staggered solution scheme has been proposed and implemented in FEAP [23] to solve the partial differential equations governing heat transfer and thermo-elasticity in the three-dimensional domain of the laminate, and then predict moisture diffusion in the two dimensional domains represented by the EVA layers and channel cracks in Silicon by considering a material diffusivity dependent on temperature and crack opening. Fractional calculus has been used to model the visco-elastic behaviour of the EVA material layer, while moisture diffusion has been assumed to be of classical type. The use of fractional derivatives in partial differential equations (PDEs) related to diffusion is also a viable modelling approach and it is usually related to a diffusion process taking place across a non-Euclidean domain, see e.g. [24]. However, due to a lack of specific experimental evidence of fractal diffusive domains, the classic PDE for diffusion has been considered in the present study.

The proposed methodology has been successfully applied to the simulation of the qualification tests required by the International Electrotechnical Commission [4], namely the *damp heat* test and the *humidity freeze* test. In the latter, due to a continuous variation of temperature during the test, we have shown that coupling between the thermo-mechanical field and moisture diffusion has to be taken into account to correctly predict the spatio-temporal evolution of moisture in the PV module. Moreover, percolation through channel cracks in Silicon solar cells has been found to significantly enhance moisture diffusion. This trend has been confirmed by experimental tests performed by the present authors and showing an increased electric degradation in cracked solar cells with respect to the intact ones.

**Fig. 19:**
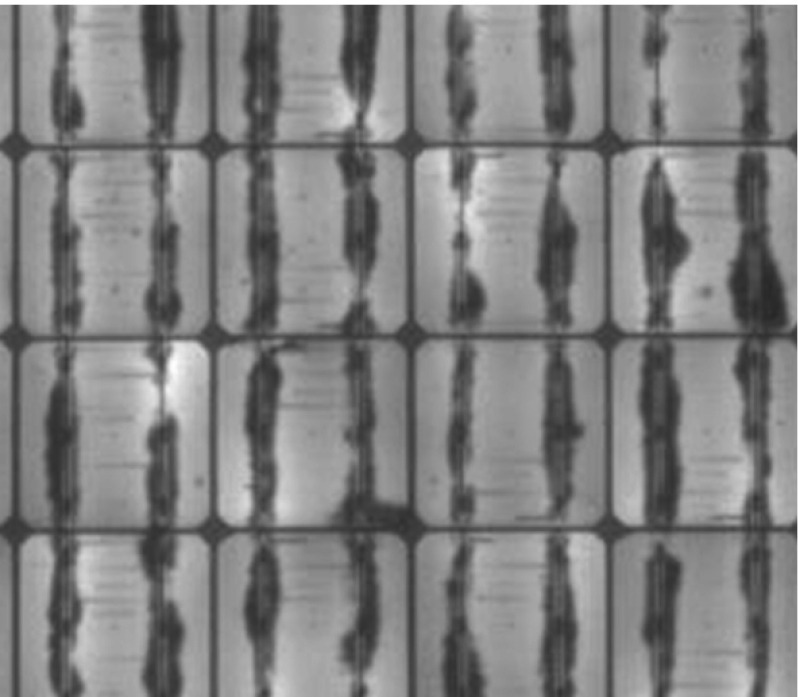
Electroluminescence image of a PV module showing moisture degradation along the busbars, due to imperfect sealing (adapted from [2])

With the present computational tool available, further developments may regard the simulation of other moisture diffusion phenomena observed in experiments, in addition to percolation through Silicon channel cracks and to diffusion along the EVA layers. As shown in [2], in fact, imperfect sealing of the region near the main electric conductors (busbars) soldered on the surface of Silicon can further enhance percolation along them, creating preferred streams for moisture diffusion and chemical oxidation, see Fig. 19. To account for this type of degradation, a possible method is to model inhomogeneous diffusivity properties inside the EVA layer above the busbars by specifying an initial separation of the EVA interface.

## Appendix

The expression of the matrices entering the discretized weak forms (23) and (24) are:

46a $$\begin{aligned}&\left[ M^{uu}_e\right] _{aIbJ} =\int _{R_e} \rho \varPhi _b \varPhi _a \mathrm {d}V \end{aligned}$$

46b $$\begin{aligned}&[K^{uu}_e]_{aIbK} =\int _{R_e} C_{IJKL} \dfrac{\partial \varPhi _b}{\partial x_L} \dfrac{\partial \varPhi _a}{\partial x_J} \mathrm {d}V \end{aligned}$$

46c $$\begin{aligned}&\left[ K^{u \theta }_e\right] _{abK} =\int _{R_e} \varPhi _a \beta \delta _{KL} \dfrac{ \partial \varPhi _b }{\partial x_L } \mathrm {d}V \end{aligned}$$

46d $$\begin{aligned}&\{ F^{u}_{e} \}_{aI}=\int _{\partial R_e} t^*_{I} \varPhi _a \mathrm {d}A \end{aligned}$$

46e $$\begin{aligned}&\left[ M^{\theta \theta }_e\right] _{ab} =\int _{R_e} \rho c \varPhi _b \varPhi _a \mathrm {d}V \end{aligned}$$

46f $$\begin{aligned}&\left[ K^{\theta \theta }_e\right] _{ab} =\int _{R_e} k \dfrac{\partial \varPhi _a }{\partial x_L } \dfrac{\partial \varPhi _b }{\partial x_L } \mathrm {d}V \end{aligned}$$

46g $$\begin{aligned}&\left[ C^{\theta u}_e\right] _{bKa} =-\int _{R_e} T_0 \beta \delta _{KL} \dfrac{ \partial \varPhi _b }{\partial x_L } \varPhi _a \mathrm {d}V \end{aligned}$$

46h $$\begin{aligned}&\left\{ F^{\theta }_e \right\} _{a}=\int _{\partial R_e} q^* \varPhi _a \mathrm {d}A \end{aligned}$$

for $1 \le a,b \le N$ and $1 \le I,J,K \le 3$. The vector $\{ f^u_e \}$ and $\{ f^{\theta }_e \}$ in components read:

47a $$\begin{aligned}&\{ f^{u}_{e} \}_{bK} =-\int _{S_e} t_{J} ([[ U_L ]] , \left\langle \varTheta \right\rangle ) \varPsi _a \Delta _{aJbK} \mathrm {d}A \end{aligned}$$

47b $$\begin{aligned}&\{ f^{\theta }_e \}_{b} =\int _{S_e} q([[ U_L ]] , \left\langle \varTheta \right\rangle ) \varPsi _a \Delta _{ab} \mathrm {d}A \end{aligned}$$

for $1 \le b \le 2S$ and $1 \le K \le 3$ where it is remarkable to note that $[C^{\theta u}_e]= -T_0 [K^{u \theta }_e]^T$. The expression of the matrices in (25) are:

48a $$\begin{aligned}&[K_e] =[P]^T \begin{bmatrix} [K^{uu}_e]&\quad \left[ K^{u \theta }_e\right] \\ 0&\quad [K^{\theta \theta }_e] \end{bmatrix}[P]&\end{aligned}$$

48b $$\begin{aligned}&[C_e] =[P]^T \begin{bmatrix} 0&\quad 0 \\ [C^{\theta u}_e]&\quad [C^{\theta \theta }_e] \end{bmatrix}[P]&\end{aligned}$$

48c $$\begin{aligned}&[M_e] =[P]^T \begin{bmatrix} \left[ M^{uu}_e\right]&\quad 0 \\ 0&\quad 0 \end{bmatrix}[P]&\end{aligned}$$

48d $$\begin{aligned}&\{ F_e \}=[P]^T \begin{pmatrix} \{ F^u_e \} \\ \{ F^{\theta }_e \} \end{pmatrix}&\end{aligned}$$

48e $$\begin{aligned}&\{ f_e \}=[P]^T \begin{pmatrix} \{ f^u_e \} \\ \{ f^{\theta }_e \} \end{pmatrix} \end{aligned}$$

The matrices entering the discretized weak form related to moisture diffusion have the following expression:

49a $$\begin{aligned}{}[B_e]_{ab}&=\int _{S_e} D \dfrac{ \partial \varPsi _a }{ \partial x_I } \dfrac{ \partial \varPsi _b }{ \partial x_I } \mathrm {d}A \end{aligned}$$

49b $$\begin{aligned}{}[A_e]_{ab}&=\int _{S_e} \varPsi _a \varPsi _b {\mathrm {d}}A \end{aligned}$$

The components of the tangent operator in Eq. (42) are:

50 $$\begin{aligned}&\left[ \dfrac{\partial \{ f^{u}_e \}_{aI}}{ \partial \{ U_e \}_{bK} } \right] ^{n+1}_{(k)} = \left[ - \dfrac{ \partial }{ U_{bK} } \int _{S_e} t_J \varPsi _a \Delta _{aJbK} \mathrm {d}A \right] ^{n+1}_{(k)}= \nonumber \\&\qquad - \int _{S_e} \varPsi _c \Delta _{cLbK} \left[ \dfrac{ \partial t_J}{\partial [[ U_L ]] } \right] ^{n+1}_{(k)} \varPsi _a \Delta _{aJbK} \mathrm {d}A\end{aligned}$$

51 $$\begin{aligned}&\left[ \dfrac{\partial \{ f^{u}_e \}_{aI}}{ \partial \{ \varTheta _e \}_{b} } \right] ^{n+1}_{(k)} = \left[ - \dfrac{ \partial }{ \varTheta _{b} } \int _{S_e} t_J \varPsi _a \Delta _{aJbK} dA \right] ^{n+1}_{(k)} =\nonumber \\&\qquad - \int _{S_e} \varPsi _k M_{kb} \left[ \dfrac{ \partial t_J}{ \partial \left\langle \varTheta \right\rangle } \right] ^{n+1}_{(k)} \varPsi _a \Delta _{aJbK} \mathrm {d}A \end{aligned}$$

52 $$\begin{aligned}&\left[ \dfrac{\partial \{ f^{\theta }_e \}_{a}}{ \partial \{ \varTheta _e \}_{b} } \right] ^{n+1}_{(k)} = \left[ \dfrac{ \partial }{ \varTheta _b } \int _{S_e} q\varPsi _d \Delta _{da} \mathrm {d}A \right] ^{n+1}_{(k)} \nonumber \\&\quad = \int _{S_e} \varPsi _k M_{kb} \left[ \dfrac{ \partial q}{ \partial \left\langle \varTheta \right\rangle } \right] ^{n+1}_{(k)} \varPsi _d \Delta _{da} \mathrm {d}A \end{aligned}$$

and

53 $$\begin{aligned} \begin{aligned}&\left[ \dfrac{\partial \{ f^{\theta }_e \}_{a}}{ \partial \{ U_e \}_{bI} } \right] ^{n+1}_{(k)} = \left[ \dfrac{ \partial }{ U_{bI} } \int _{S_e} q \varPsi _d \Delta _{da} dA \right] ^{n+1}_{(k)} \\&\quad = \int _{S_e} \varPsi _i \Delta _{iLbI} \left[ \dfrac{ \partial q}{ \partial [[ U_L ]] } \right] ^{n+1}_{(k)} \varPsi _d \Delta _{da} \mathrm {d}A \end{aligned} \end{aligned}$$

where:

54a $$\begin{aligned}&\left[ \dfrac{ \partial t_J}{\partial [[ U_L ]] } \right] = \left[ \begin{array}{lll} K_1(t,\left\langle \varTheta \right\rangle ) \chi _1 &{}\quad 0 &{}\quad 0 \\ 0 &{}\quad K_2(t,\left\langle \varTheta \right\rangle )\chi _2 &{} \quad 0 \\ 0 &{}\quad 0 &{}\quad K_3(t,\left\langle \varTheta \right\rangle )\chi _3 \end{array}\right] \end{aligned}$$

54b $$\begin{aligned}&\left[ \dfrac{ \partial t_I}{ \partial \left\langle \varTheta \right\rangle } \right] =\frac{ \partial }{\partial \left\langle \varTheta \right\rangle }(K_I(t,\left\langle \varTheta \right\rangle ))\chi _I \end{aligned}$$

54c $$\begin{aligned}&\left[ \dfrac{ \partial q}{ \partial \left\langle \varTheta \right\rangle } \right] =-k_0 \left( 1- \dfrac{[[ U_3 ]]}{\delta ^c_3} \right) \chi _3 \end{aligned}$$

54d $$\begin{aligned}&\left[ \dfrac{ \partial q}{ \partial [[ U_L ]] } \right] =\left( \begin{array}{ll} &{} 0 \\ &{} 0 \\ &{} -k_0 \left( 1- \dfrac{1}{\delta ^c_3} \right) \left\langle \varTheta \right\rangle \ \chi _3 \end{array}\right) \end{aligned}$$

$\chi _I$ ($1 \le I \le 3$) being the characteristic function such that $\chi _I=\chi _{ \left\{ [[ U_I ]] \in J_I \right\} }$.

$[M]_{ab}$ for $(1 \le a \le S(e), 1 \le b \le 2 S(e))$ is the mean operator computing the average temperature across the EVA layer, i.e.

55 $$\begin{aligned}{}[M_e]_{ab} \varTheta _b=\left\langle \varTheta _b \right\rangle . \end{aligned}$$
